# Intermediate term follow-up after a single-piece-acrylic intraocular lens implantation in the ciliary sulcus- a cross-sectional study

**DOI:** 10.1186/1471-2415-13-76

**Published:** 2013-12-09

**Authors:** Shai M Bar-Sela, Efrat Fleissig

**Affiliations:** 1 Department of Ophthalmology, Tel Aviv Medical Center, Sackler Faculty of Medicine, Tel Aviv University, Tel Aviv, Israel

**Keywords:** Cataract, Intraocular lens, Posterior capsule tear

## Abstract

**Background:**

Implantation of a single-piece-acrylic intraocular lens (SPA-IOL) in the ciliary sulcus during phacoemulsification complicated with posterior capsule tear (PCT) may be associated with severe complications. The purpose of this study was to report the efficacy and safety of sulcus implantation of a SPA-IOL, designed for both in-the-bag and sulcus positioning.

**Methods:**

A prospective cross-sectional study including 12 patients, who underwent phacoemulsification with PCT and sulcus implantation of SPA-IOL designed for both in-the-bag and sulcus positioning (Seelens AF, Hanita, Israel) between January 2009 and March 2012 (follow-up 12–37 months). Preoperatively corrected distance visual acuity (CDVA), subjective refraction and intraocular pressure (IOP) were recorded. Postoperative evaluation included anamnesis for IOL edge symptoms and transient visual obscurations (TVO) along with CDVA, subjective refraction IOP, anterior segment biomicroscopy, gonioscopy, assessment of IOL centration, fundus biomicroscopy and spectral-domain optical coherence tomography of the macula.

**Results:**

Preoperatively, mean CDVA was 0.84 ± 0.60 LogMAR (Counting Finger-20/33) improving to 0.18 ± 0.13 LogMAR (20/40-20/20) at last examination (p = 0.004), as all the patients gained better CDVA. Mean preoperative spherical equivalent was −0.2 ± 2.5 Diopter (D) (−4.0D to +5.4D) reaching −1.9 ± 0.9 (−4.0D to −0.6D) at last examination (p = 0.12). Mean preoperative refractive astigmatism magnitude was 1.0 ± 0.6D (0.3D to 2.3D) changing to 1.1 ± 1.0D (0.0D to 3.0D) at last examination (p = 0.88). Mean preoperative IOP was 14.7 ± 3.2 mmHg (11–23 mmHg) without medications reaching 15.9 ± 3.3 mmHg (10–21 mmHg) at last follow up (p = 0.21). Postoperatively one patient required two medications for IOP control in his study and contralateral eyes. None of the patients had symptoms of IOL edge or TVO. There were no intraocular hemorrhages, inflammatory reactions, or pigment dispersion and the IOLs were well centered in all cases. Central foveal thickness was 280 ± 33 μm (193–310 μm).

**Conclusions:**

Appropriately designed SPA-IOL may be implanted in the ciliary sulcus during phacoemulsification with PCT rather than switching to another backup IOL demanding wound enlargement.

## Background

Posterior capsule tear (PCT) is a common complication during phacoemulsification with reported incidence ranging from 0.7% to 16%, which may preclude intraocular lens (IOL) implantation in the capsular bag [1]. Yet, in some cases IOL may be fixated in the ciliary sulcus if there is sufficient support of the residual capsule structure [1].

Single-piece-acrylic (SPA) IOLs have been proved to be excellent posterior chamber IOLs (PCIOLs) when placed in the lens capsular bag. However, several studies have shown that ciliary sulcus implantation of SPA-IOLs designed solely for capsular bag placement may result in severe complications [2-8] such as pigment dispersion syndrome (PDS), secondary intraocular pressure (IOP) elevation, IOL decentration with edge symptoms, intraocular hemorrhages, recurrent iridocyclitis and cystoid macular edema.

The purpose of this cross-sectional study was to evaluate the long term follow-up after sulcus fixation of a SPA-IOL (SeeLens AF, Hanita Lenses, Kibbutz Hanita, Israel) with appropriate design for implantation in the capsular bag and the ciliary sulcus.

## Methods

We reviewed the medical records of all patients who underwent phacoemulsification complicated with PCT between January 1, 2009 and March 31, 2012 in the Tel Aviv Medical Center, the tertiary Hospital of the Tel Aviv metropolitan area, affiliated with Tel Aviv University, Israel.

After approval of this cross-sectional-analysis by the Investigational Review Board of the Tel Aviv Medical Center 12 patients, with SPA-IOL implanted in the ciliary sulcus, were included in this study. All patients gave their informed consent before being included in the study.

The following preoperative data was retrieved from the patients’ medical records: age, sex, ocular comorbidity, biometric data, corrected distance visual acuity (CDVA), subjective refraction and IOP.

Surgery was performed using topical or peribulbar anesthesia. Two paracentesis ports were opened at the limbus at the 2 and 10 o’clock positions, and an anterior chamber maintainer was inserted through another paracentesis port located inferiorly. Capsulorrhexis was performed using a bent 25-gauge needle or forceps and a superotemporal, near-limbus, corneal main incision of 2.4 mm was created. Following hydrodissection, phacoemulsification of the nucleus was performed with the Infiniti Vision or the Legacy Systems (Alcon Surgical) using the divide-and-conquer [9] or the stop-and-chop [10] techniques, and the cortex was removed using an aspirating cannula. When posterior capsule rupture was noticed, the surgical technique was changed so to avoid or minimize vitreous loss by maintaining a closed intraocular system with stable pressure and by injection of ophthalmic viscosurgical device (OVD) to tamponade the capsule rupture. Nuclear remnants were removed using phacoemulsification with decreased flow, aspiration and vacuum rate and cortical remnants were cleaned with an aspirating cannula. Anterior vitrectomy was performed if vitreous loss was observed.

**Figure 1:**
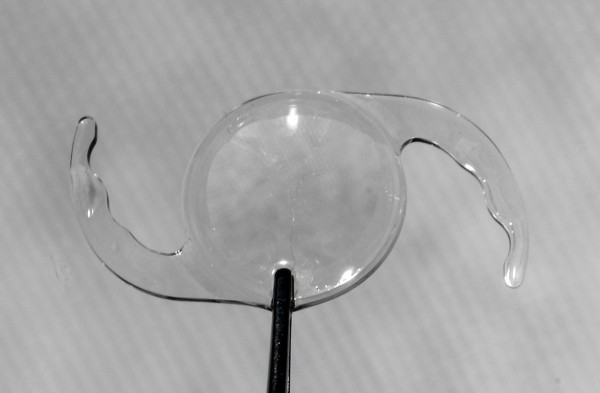
The single-piece-acrylic intraocular lens used in this study.

**Figure 2:**
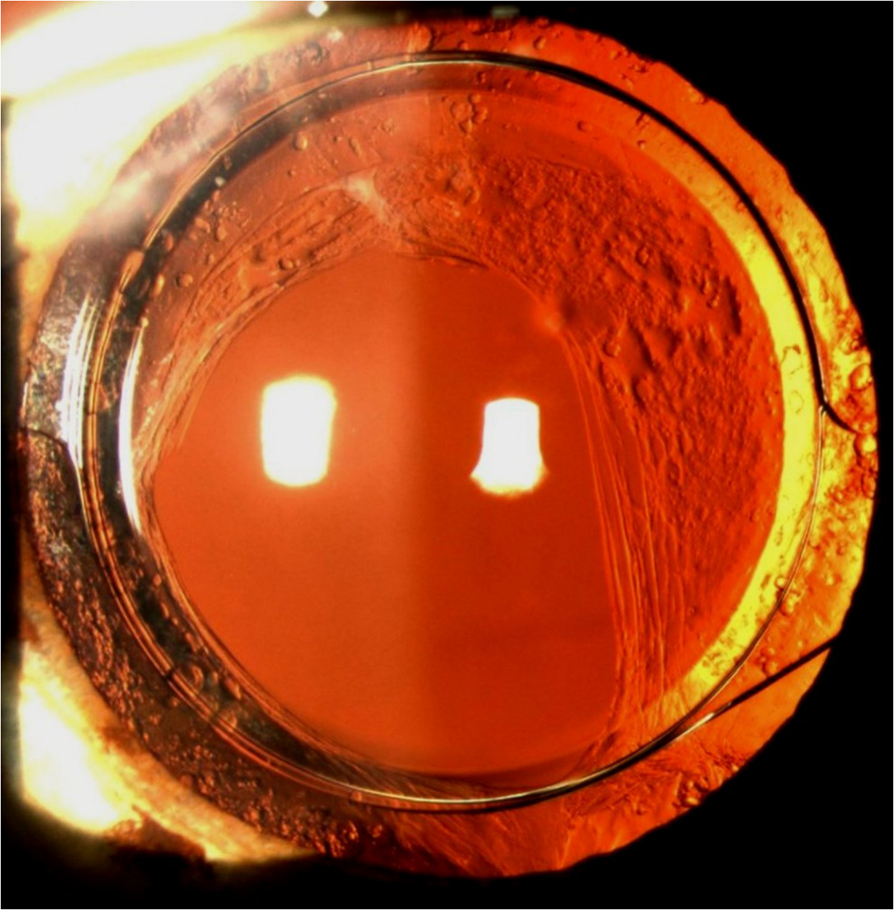
**A slit lamp photograph of a single-piece-acrylic intraocular lens implanted in the ciliary sulcus.** A representative slit lamp photograph of the right eye 29 months after phacoemulsification complicated with large posterior capsule tear and vitreous loss. The eye is quiet; a single-piece-acrylic intraocular lens is symmetrically centered in the ciliary sulcus with the haptics, the optic-haptic junctions and the optic edge supported by the anterior capsule.

Following an evaluation of sufficient capsule support for IOL fixation in the ciliary sulcus, an OVD was injected in the anterior chamber and between the anterior capsule and the posterior iris. Then a SPA foldable PCIOL with 6.0 mm optic, 13.0 mm overall length, 5-degree posterior angulation, C-loop design and haptic thickness of 0.31 mm (SeeLens AF, Hanita Lenses, Kibbutz Hanita, Israel; Figure 1; Additional files 1 and 2) was implanted through the 2.4 mm incision using an injector. The IOL was first guided and unfolded in the anterior chamber. Then using a Sinskey hook the haptics were placed in the ciliary sulcus and when necessary the IOL was rotated according to the capsule remnants for maximal support (Figure 2). At the end of the surgery miochol was injected to constrict the pupil and assure that no vitreous was prolapsed to the anterior chamber, then the OVD was aspirated and the corneal wound was closed with hydration without suture application.

Postoperative treatment included topical steroids, antibiotics and non-steroidal-anti-inflammatory agents according to the surgeon’s preference. Usually the patients were followed at the cataract clinic of our Medical Center during the first postoperative month and then sent for continued care at the community-based-clinics of their health maintenance organization.

After a postoperative period of at least 1 year the patients included in this prospective cross-sectional study were invited for anamnesis evaluating IOL edge symptoms and transient visual obscurations (TVO) along with the following examinations: Snellen corrected distance visual acuity (CDVA), subjective refraction, anterior segment biomicroscopy, gonioscopy, IOP, assessment of IOL centration, fundus biomicroscopy and spectral domain optical coherence tomography (SD-OCT) (Spectralis; Heidelberg Engineering, Heidelberg, Germany) of the macula.

IOL centration was evaluated after pupil dilation with Tropicamide 0.5% and Phenylephrine Hydrochloride 10% using a method described elsewhere [11]. Our 6.0 mm optic diameter IOL was defined as decentered when its optic edge was visible through 5.0 mm diameter pupil as a result of tilt or subluxation greater than 1.0 mm.

The CDVA results were converted from Snellen to logMAR notation for statistical analysis. The paired t –test was used to compare between the preoperative and the postoperative results of the spherical equivalent (SEQ), refractive astigmatism, CDVA and IOP, and a *P* value less than 0.05 was considered significant.

## Results

Twelve patients, (five men and seven women) were included in the cross-sectional study with mean age of 67 years (range, 50 to 85 years) and mean follow-up period of 25 months (range, 12 to 37 months).

Ocular comorbidity included three patients with regressed diabetic macular edema (DME) and regressed proliferative diabetic retinopathy (PDR) preoperatively treated with focal laser and scatter laser, respectively; one of them had optic atrophy as well.

The mean axial length was 23.27 ± 0.87 mm (22.15 to 24.70 mm), the mean keratometry (K) reading was 44.6 ± 1.8 Diopter (D) (42.35 to 49.02D) and the mean power of the implanted IOL was 21.3D (15.5 to 26.0D).

Preoperatively, mean CDVA was 20/138 [0.84 ± 0.60 LogMAR; Counting Finger (CF) to 20/33] improving to 20/30 (0.18 ± 0.13 LogMAR; 20/40 to 20/20) at last examination (p = 0.004), as all the patients gained better CDVA. Five patients out of twelve had final CDVA lower than 20/30: two patients had regressed DME and regressed PDR, one of whom had optic atrophy as well; the third had thick epiretinal membrane (ERM), vitreo-macular traction (VMT) syndrome and CME; the forth had stromal corneal opacity; the fifth had posterior capsule opacity, awaiting posterior capsulotomy.

Preoperative refraction was recorded in 10 of the 12 patients included in this study; 2 patients had brunescent cataract and preoperative CDVA of CF interfering with accurate refraction measurements.

The Mean preoperative spherical equivalent was −0.2 ± 2.5D (−4.0D to +5.4D) changing to −1.9 ± 0.9 (−4.0D to −0.6D) at last examination (p = 0.12). The mean preoperative refractive astigmatism magnitude was 1.0 ± 0.6D (0.3D to 2.3D) reaching 1.1 ± 1.0D (0.0D to 3.0D) at last examination (p = 0.88).

One patient with brunescent cataract and preoperative CDVA of CF, precluding preoperative subjective astigmatism magnitude measurement, had a SEQ of −4.0D and refractive astigmatism of 3D at last follow-up. Two patients with preoperative corneal cylinder of 2.3D and 1.9D had a refractive astigmatism magnitude of 2.5D and 1.75D, respectively, at last follow-up.

The remaining patients had postoperative SEQ and refractive cylinder magnitude lower than 2.4D and 1.25D, respectively, at last follow-up.

Mean preoperative IOP was 14.7 ± 3.2 mmHg (range, 11 to 23 mmHg) without medications reaching 15.9 ± 3.3 mmHg (range, 10 to 21 mmHg) at last follow up (p = 0.21). During the postoperative follow-up period one patient developed glaucoma in both the study and the contralateral eyes requiring two medications for IOP control.

None of the patients had symptoms of IOL edge glare or TVO.

**Figure 3:**
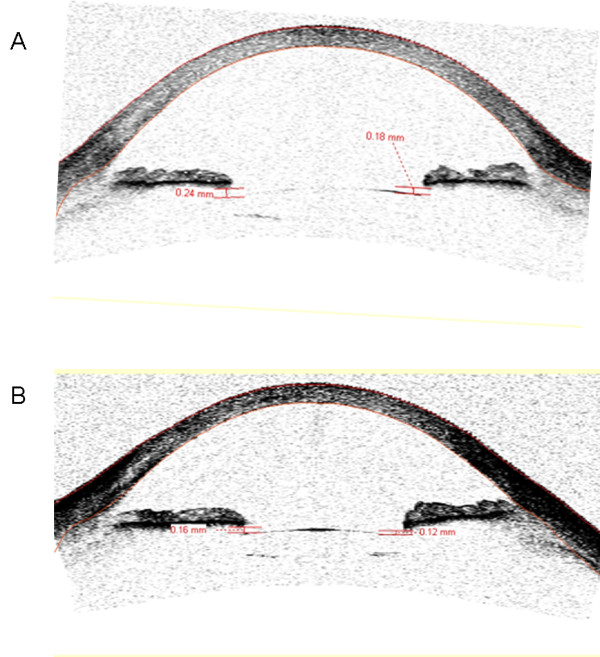
**Anterior segment OCT images of a single-piece-acrylic intraocular lens implanted in the ciliary sulcus.** Horizontal **(A)** and vertical **(B)** anterior segment OCT (Visante™OCT, Carl Zeiss Meditec, Inc., CA, USA), images of the right eye with sulcus-fixated single-piece-acrylic intraocular lens (IOL), 6 months after phacoemulsification complicated with large posterior capsule tear and vitreous loss. The similar distance between the anterior edge of the IOL and the posterior iris, indicates that there is no optic tilt.

On slit lamp examination there were no cells, flare, hemorrhage or vitreous incarceration in the anterior chamber. In addition there were no pigment depositions on the cornea or the IOL and the IOLs were well centered in all patients (Figures 2 and 3). Gonioscopy revealed open angles with similar trabecular meshwork pigmentation in each study eye compared to the contralateral eye.

None of the patients had vitreal cells or hemorrhage. Clinical retinal examinations and SD-OCT revealed flat maculas without hydration or cysts, except for one patient who had thick ERM, VMT and CME. Accordingly, the mean postoperative central foveal thickness measured by SD-OCT was 280 ± 33 μm (range, 193 to 310 μm).

## Discussion

Our results show that sulcus fixation of a SPA-IOL, designed for both intracapsular and sulcus implantation, may be safe and efficient as it resulted in significant improvement of the mean postoperative CDVA compared to the preoperative records (20/30 versus 20/138; p = 0.004) without the associated complications reported with sulcus fixation of other SPA-IOLs designed exclusively for implantation in the capsular bag.

Pseudophakic PDS is the most common problem within this group of complications. It is characterized by increased pigmentation on the anterior surface of the IOL, the corneal endothelial surface and the trabecular meshwork and assumed to result from contact between the PCIOL and the posterior iris leading to chafing of the posterior iris pigment. Therefore it has been mostly reported with sulcus fixated PCIOLs positioned in close proximity to the posterior iris than with PCIOLs implanted in the capsular bag [12-15].

Moreover, sulcus placement of SPA-PCIOL designed solely for capsular bag implantation may further increase the risk of this complication [2-8]. It is thought that several structural elements of these IOLs are responsible for this finding [1,16]: 1. their haptics are thick and bulky, allowing contact with the iris surface when placed in the ciliary sulcus; 2. their shape is planar rather than posteriorly angulated and, therefore, the optic does not vault posteriorly from the iris; 3. their overall diameter from haptic to haptic is shorter than the sulcus diameter, enabling lateral decentration of the IOL with further proximity to the posterior iris.

Unlike these IOLs, the SPA-PCIOL used in our study was appropriate for ciliary sulcus implantation owing to several features: 1.its haptics are thin (0.31 mm), minimizing the contact with the posterior iris surface; 2. its 5-degree posterior haptics angulation moves the optic away from the iris, thus reducing iris chafing by both haptics and optics and providing sufficient posterior iris clearance when placed in the ciliary sulcus; 3. its large overall diameter of 13.0 mm may minimize the lens movement [1], which can increase posterior iris chafing. These unique features may explain the absence of PDS in our patients. However, more data is required to confirm our observations.

Moreover, the pigment accumulating in the trabecular meshwork may restrict aqueous outflow leading to secondary pigment dispersion glaucoma (PDG) [2,6,7]. Uy and Chan [6] reported PDG in 3 of their patients (15%) after SPA-PCIOL implantation in the ciliary sulcus. As opposed, only one of our patients developed open angle glaucoma postoperatively in both the study and the contralateral eyes, without the typical PDG signs of pigment scatter in the anterior chamber. The difference between our study and the other reports may be explained by the unique characters of the SPA-PCIOL used in this study, minimizing iris chafing, pigment liberation and the consequent PDG.

Furthermore, PCIOL positioned in the sulcus in close contact with the iris or the ciliary body may erode the uveal vasculature causing vitreous hemorrhage [3-5] or may break the blood–aqueous barrier leading to the uveitis-glaucoma-hyphema (UGH) syndrome [8]. This syndrome is composed of a spectrum of disorders including pigment dispersion with or without intraocular pressure increase, intermittent hyphema with TVO, or uveitis with or without cystoid macular edema. Unlike these reports, none of our patients had postoperative intraocular hemorrhage, inflammatory reaction, or symptoms of TVO. This discrepancy may be elucidated by the structure of our IOL decreasing its contact with the iris, but larger case series are needed to validate this assumption.

Moreover, our clinical findings showing no inflammatory reaction were supported by macular SD-OCT revealing absence of CME in all of our patients except for one who had severe vitreo-retinal pathology leading to macular traction and CME. However, more studies using SD-OCT are required to validate our findings.

Another demand for any sulcus implanted IOLs is to keep its central and steady positioning as decentered IOL with its optic edge visible within the pupillary space may result in glare symptoms [16]. Moreover, decentration of sulcus fixated IOL may accentuate iris scraping and breakdown of the blood–aqueous barrier leading to pigment dispersion and UGH [16] and may induce myopia and astigmatism [17].

Brazitikos et al. [11] reported decentration of 1.0 to 2.0 mm in 5 cases (17.9%) after 3-piece acrylic PCIOL positioned in the sulcus. Therefore, an overall diameter of 13.0 mm has been recommended for sulcus fixated IOLs to prevent subluxation and unexpected lens movement, in addition to optic diameter of 6.0 mm to avoid glare symptoms in case of mild postoperative decentration [1].

As opposed, biomicroscopic examination with dilated pupils did not reveal IOL decentration greater than 1.0 mm in any of our patients, and none of them had glare symptoms. These observations may be explained by sufficient capsule structure keeping the PCIOL positioning in the ciliary sulcus (Figure 1), and by the large diameter of the IOL used in this study [1].

However, one of our patients had significant postoperative myopia and astigmatism that may result from IOL decentration smaller than 1 mm, which could not have been discovered by the clinical examination used in this study. On the other hand, De Castro et al. showed that Scheimpflug Tomography Imaging system (Galilei) may be valuable for demonstrating IOL decentration much smaller than 1 mm [18]. Therefore, future studies using this imaging modality are warranted to reveal the exact anatomic positioning of sulcus fixated IOL’s.

The SPA-IOL used in this study was designed for both in-the-bag and sulcus implantation through 2.4 mm wound. This finding may have significant consequences since it enables sulcus fixation of the original IOL model selected for intracapsular implantation through the existing wound instead of switching to another backup IOLs demanding wound enlargement.

## Conclusions

Sulcus implantation of PCIOL during phacoemulsification surgery complicated with PCT may be considered if adequate lens is available and sufficient capsular support exists. Our intermediate term follow-up after sulcus fixation of a SPA-IOL designed for both intracapular and sulcus implantation revealed good final CDVA and low level of complications, though more research to ascertain our results is warranted.

## Abbreviations

SPA-IOL: Single-piece-acrylic intraocular lens; PCT: Posterior capsule tear; CDVA: Corrected distance visual acuity; IOP: Intraocular pressure; TVO: Transient visual obscurations; IOL: Intraocular lens; PDS: Pigment dispersion syndrome; OVD: Ophthalmic viscosurgical device; SD-OCT: Spectral domain optical coherence tomography; ERM: Epiretinal membrane; VMT syndrome: Vitreo-macular traction; CME: Cystoid macular edema; PDG: Pigment dispersion glaucoma; UGH: Uveitis-glaucoma-hyphema; DME: Diabetic macular edema; PDR: Proliferative diabetic retinopathy.

## Competing interests

The authors declare that they have no competing interests.

## Authors’ contributions

SB; have made substantial contributions to conception and design, acquisition of data and analysis and interpretation of data; have been involved in drafting the manuscript and revising it critically for important intellectual content; and have given final approval of the version to be published. EF: have made substantial contributions to acquisition of data; have been involved in revising the manuscript; have given final approval of the version to be published.

## Pre-publication history

The pre-publication history for this paper can be accessed here:

http://www.biomedcentral.com/1471-2415/13/76/prepub

## Supplementary Material

Additional file 1 **SeeLens clinical evaluation.** Description of data: an official publication of Hanita Lenses presented at the microsurgery of the eye convention In memory of Prof. Blumenthal, Eilat, 2009. Click here for file

Additional file 2 **SeeLens intraocular lens clinical evaluation.** Description of data: an official publication of Hanita Lenses reporting clinical evaluation of SeeLens intraocular lens after 2-year follow-up, published in April 2010. Click here for file
