# Supplementary material for: Intermediate term follow-up after a single-piece-acrylic intraocular lens implantation in the ciliary sulcus- a cross-sectional study
Source: BMC Ophthalmol. 2013 Dec 9;13:76. doi: 10.1186/1471-2415-13-76 (PMC4098571; doi:10.1186/1471-2415-13-76)
Supplement: Additional file 1 — SeeLens clinical evaluation. Description of data: an official publication of Hanita Lenses presented at the microsurgery of the eye convention In memory of Prof. Blumenthal, Eilat, 2009. [file 1471-2415-13-76-S1.pdf]

SeeLens
Clinical Evaluation

Hanita Lenses

Hanita Lenses

By:

Prof. Hanna Garzozi

Bnei-Zion MC

The microsurgery of the eye convention
In memory of Prof. Blumenthal

Eilat,2009

Hanita Lenses

Background

- The SeeLens has a *CE* mark since December 2006.
- More than 10,000 SeeLens Intraocular lenses were sold across Europe and the rest of the world until October 2008.
- Excellent reviews and feedbacks were received by surgeons and distributors in all countries where the SeeLens was implanted.

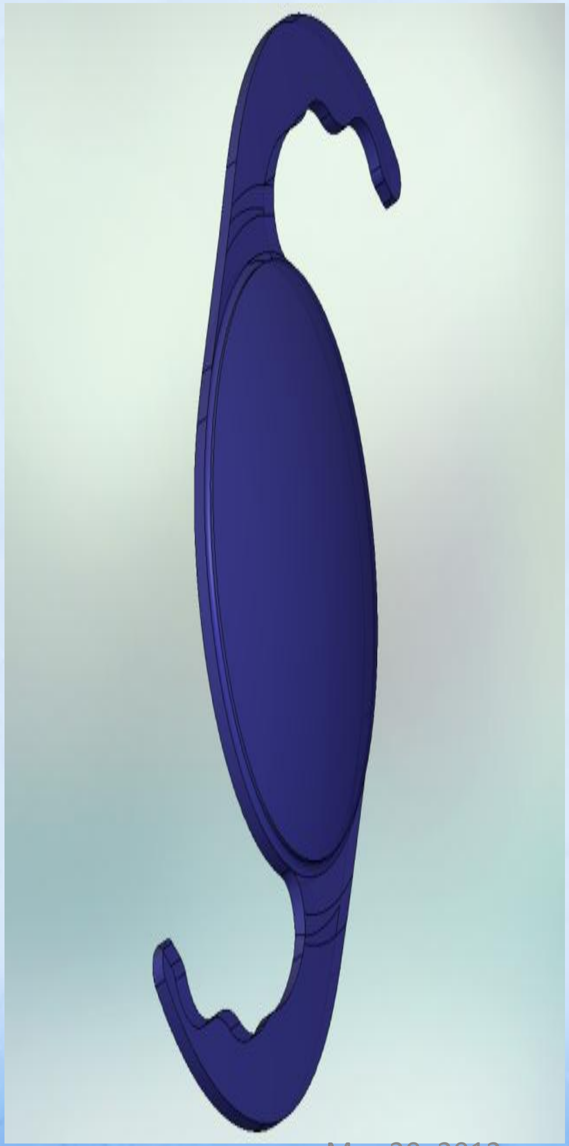

Hanita Lenses

Background

- The SeeLens was registered for sell in Israel on May 2008 with no limitations.

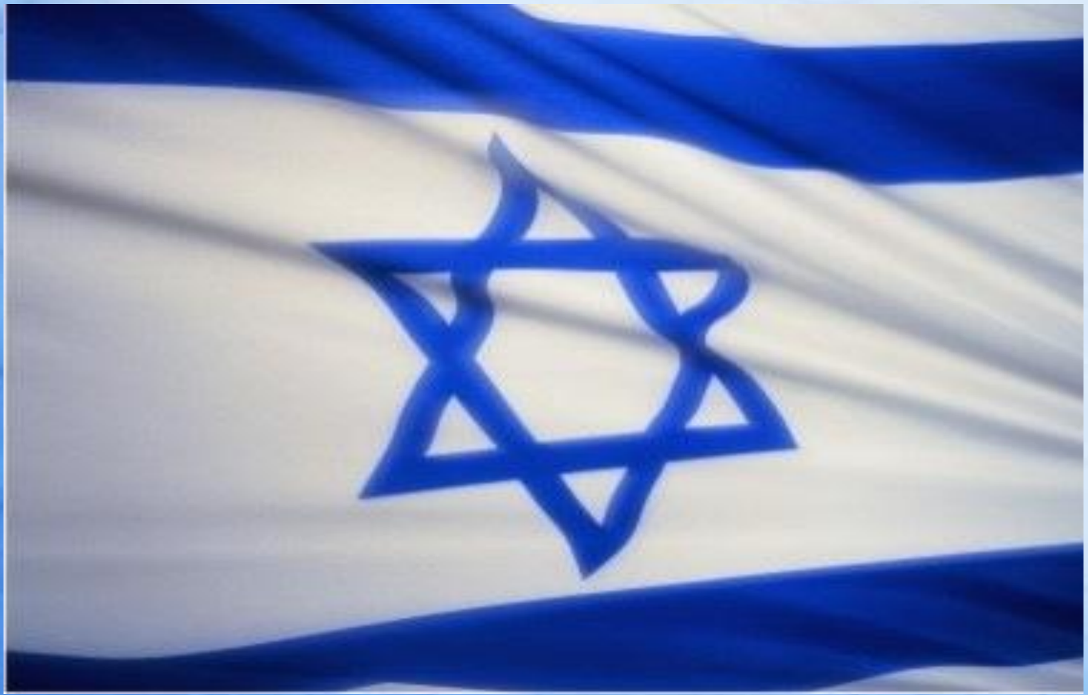

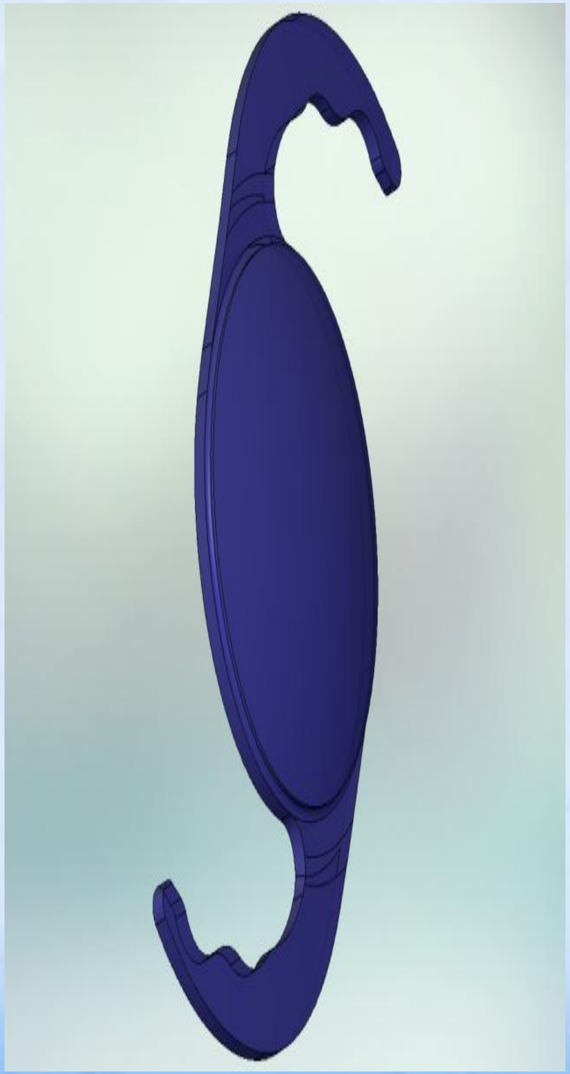

SeeLens

Specifications

Optic Diameter	6.0 mm
Overall diameter	13.0 mm
Power range	+1.0D to +40.0D<br>(7.0D to 31.0D increments of 0.5D)
Optic design	Spherical equi-convex
Lens design	Double square edge with 360 º<br>stepped barrier
Haptic angulation	5º
Material	Hydrophilic Acrylic – HEMA/EOEMA<br>UV Blocker
Refractive index	1.462 (35º c)
YAG laser	Compatible
A constant	118.6
Placement	Capsular bag or Sulcus
Injection – incision size	2.5 mm Incision

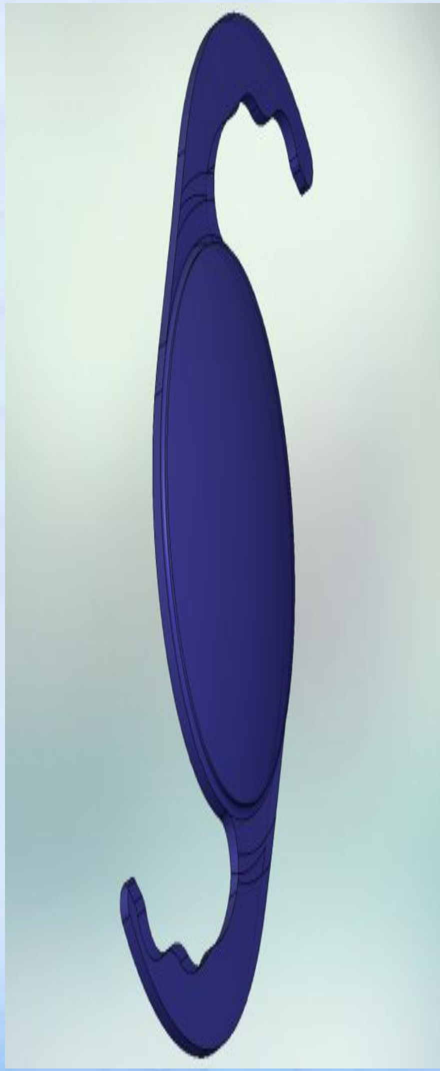

SeeLens

Square edge Design

Hanita Lenses

❖The SeeLens has a unique 360^0 double square edge

❖In order to prevent Posterior Chamaber Opacification

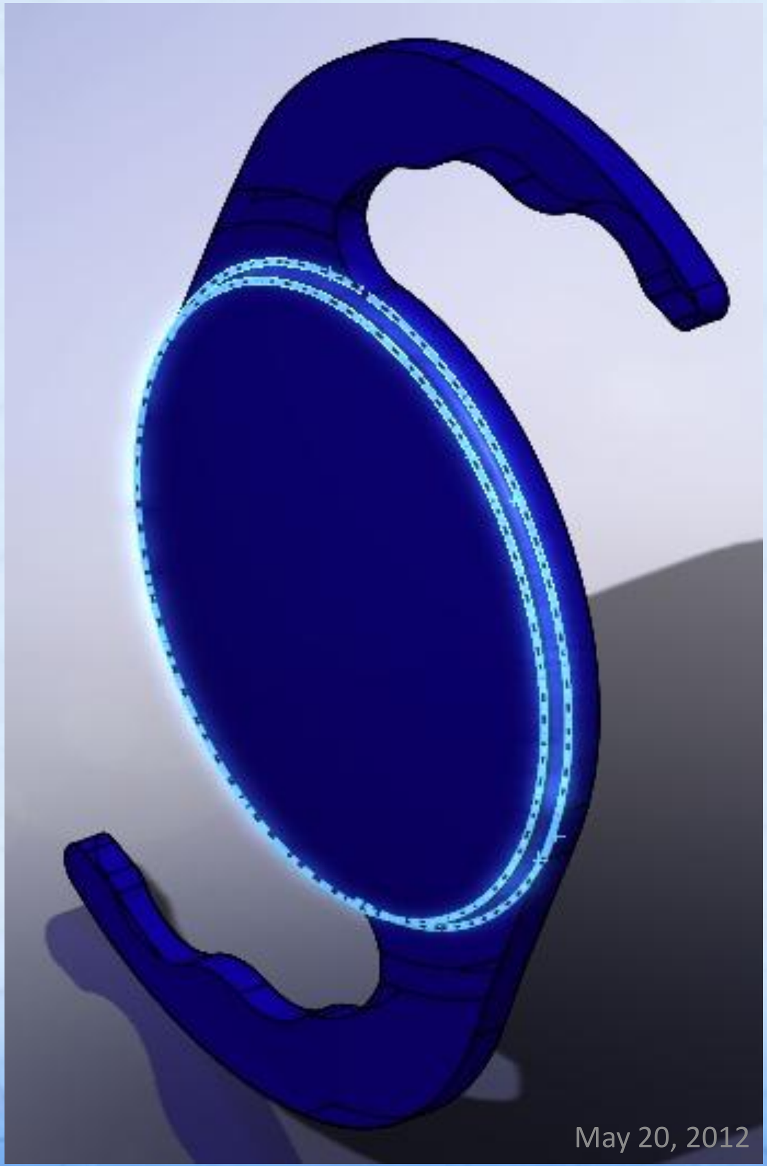

Hanita Lenses

Haptic Design

The SeeLens holds a special designed C-loop providing:

✓ Excellent stability in the capsular bag

✓Endurance to tilt & decentration.

✓Wide angle of contact with the capsular bag.

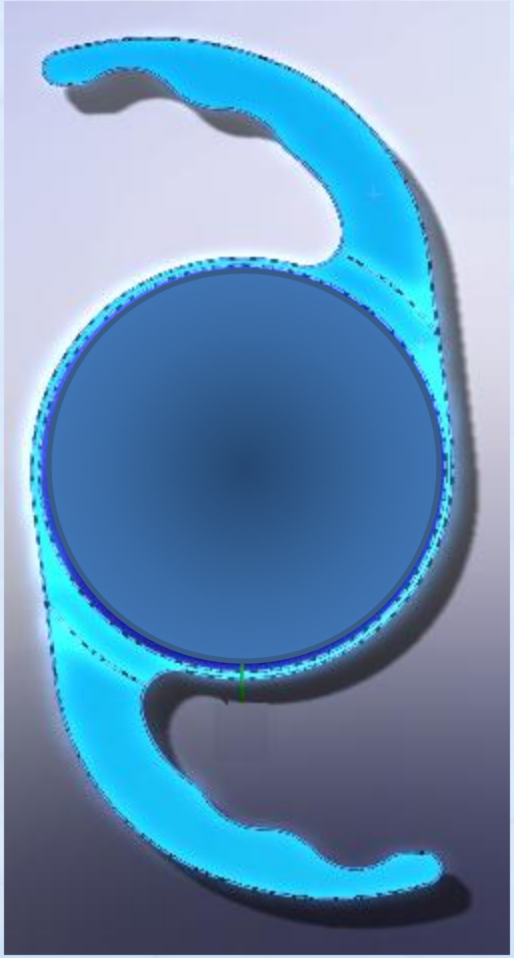

Hanita Lenses

Haptic Design

The SeeLens Haptic design has passed all the tests required by the ISO11979-3 tests.
The tests were conducted by the most distinguished and strict ophthalmic medical device test laboratory of MDT GMBH.
(I.E. Compression force, Axial length displacement , Decentration, Tilt, Angle of contact, etc.)

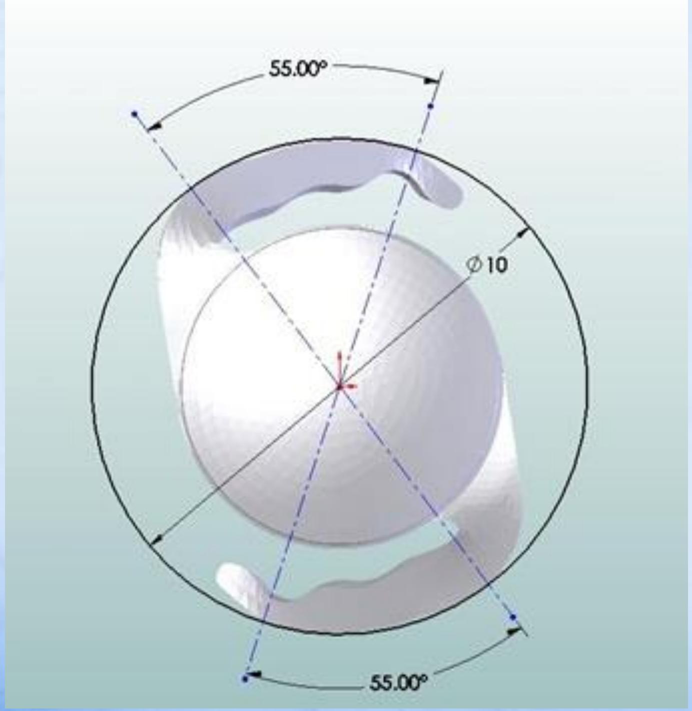

Hanita Lenses

SeeLens Evaluation

A non-randomized, open, prospective, multicenter study was conducted.

Site	Surgeon	# of patients
Ein-Tal, Israel	Prof. Ehud Assia	30
Bnei-Zion, Israel	Prof. Hana Garzuzi	33
Assuta P-T, Israel	Dr. Dan Zaksh	20
Pradubice, Czech	Prof. Jan Novak	50

Total number of patients: 133

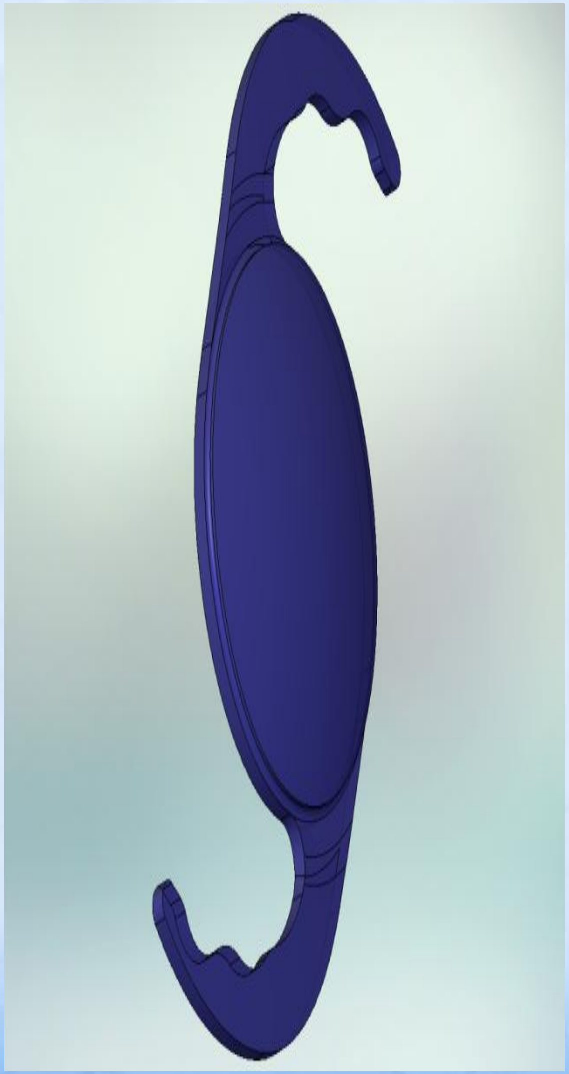

SeeLens Evaluation

Hanita Lenses

Patients follow up:

1) Pre operative examination
2) 1 day postoperatively
3) 7-10 days postoperatively
4) 1 month postoperatively
5) 3 months postoperatively.
6) 1 year postoperatively*.
7) 2 year postoperatively.

*1 year follow up started on November 2008.

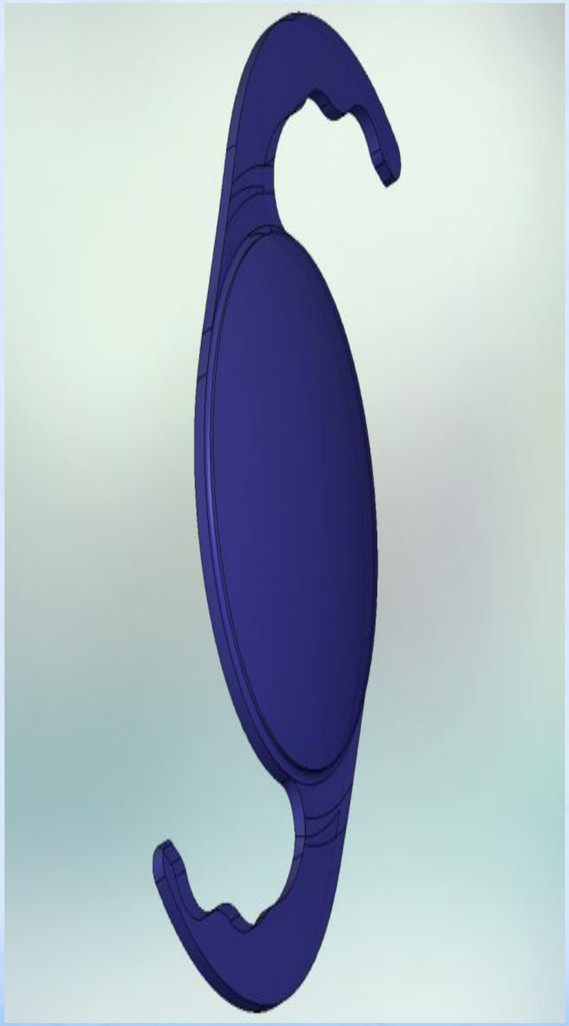

Hanita Lenses

SeeLens Evaluation

Hanita Lenses

Post operative tests:

✓Uncorrected &Best-corrected visual acuity (except for day 1)
✓Refraction.
✓Intraocular pressure.
✓Slit lamp examination
(The same tests performed preoperatively)

✓IOL location and centration.
✓Records of complication and Adverse Events
✓Extent of PCO

Eligibility criteria

INCLUSION CRITERIA

- Age > 50 years
- Senile cataract
- Refractive cylinder < -1.5 diopter
- Planed surgery technique: cataract extraction by ECCE Phacoemolsification.
- IOL power, calculated by biometry measurements by IOLMASTER, will be in the range of 15 - 25.

Hanita Lenses

Eligibility criteria

EXCLUSION CRITERIA

•**Allergy or intolerance to required study medications** (including antibiotic).
•**Amblyopia.**
•**History or evidence of any Ocular disease that may effect Visual Acuity** (i.e.: Uncontrolled glaucoma, Ocular injury, Corneal pathologies, Retinal pathologies in general and macular pathologies in particular, diabetic Retinopathy, Uveitis, Aniridia or iris atrophy, Vitreous pathologies (patients with vitreous separation or floaters can be included).
•**Rubella cataract.**
•Any other ocular condition that may predispose a subject to future complications or contraindicate implantation of the model SEELENS
•**Previous ocular surgery**, including refraction surgery (study eye).
•**Microphthalmos.**
•**Extreme axial length** (outside the range of 21.5 – 25).
•**Surgical complication (not IOL related),** that may effects study results (i.e. Capsular tear or rupture during cataract extraction).
•**Posterior Capsule scar or opacification** if demonstrated during the cataract extraction surgery.
•**Multiple operative procedures during cataract extraction**.

Hanita Lenses

ISO standards

The ISO Standard:
Requirements defined by ISO 11979-7 2001.

1. Post Operative BCVA of at least 6/12 (20/40) within 88% of patients' population.

2. IOL related Post Operative complication and Adverse Events equal to or less then the allowed rate defined by ISO 11979-7 2001.

Hanita Lenses

Evaluation Key Parameters

Success criteria for the study were chosen according to the ISO standards .
More efficacy Hanita Lenses .

❖ **The key efficacy parameters are:**

Best Corrected Visual Acuity (BCVA)
Predictability of refractive correction

❖ **The key safety parameters include:**

IOL behavior during implantation and follow-up
IOL related infection and/or inflammatory reactions
Posterior Capsular Opacification (PCO).

Hanita Lenses

3month follow up

❖The post operative statistics was performed on the 3 month follow up.

❖Paired T-test was done on the post op 1 day 1 week, 1 month, 3 month surveys.

❖ANOVA (Analysis of Variance) was done to the different groups of surgeons in order to determine the parameters that might be affiliated to the surgeon style and technique on the lens performance.

Preoperative General Data

Age: 71±8, range- 51~90
Gender: female 77 (57.9%) male 56 (42.1%)
Eye: left 67 (50.4%) right 66 (49.6%)
Ocular disease: 20 (15%)
i.e: hypermetropia, glaucoma, IFIS, AMD.
Pseudoexfoliation:
scaled from 0(no PXF)- 4 (hard PXF)

PXF Scale	0	1	2	3	4
Sum<br>percent	123<br>92.5%	2<br>1.5%	7<br>5.3%	1<br>0.8%	0<br>0%

Systemic disease: 84 (63.2%)
mostly hypertension and heart problem

SeeLens Clinical Evaluation

Implantation Behavior

Scale : 0(Excellent performance)-4(poor performance)

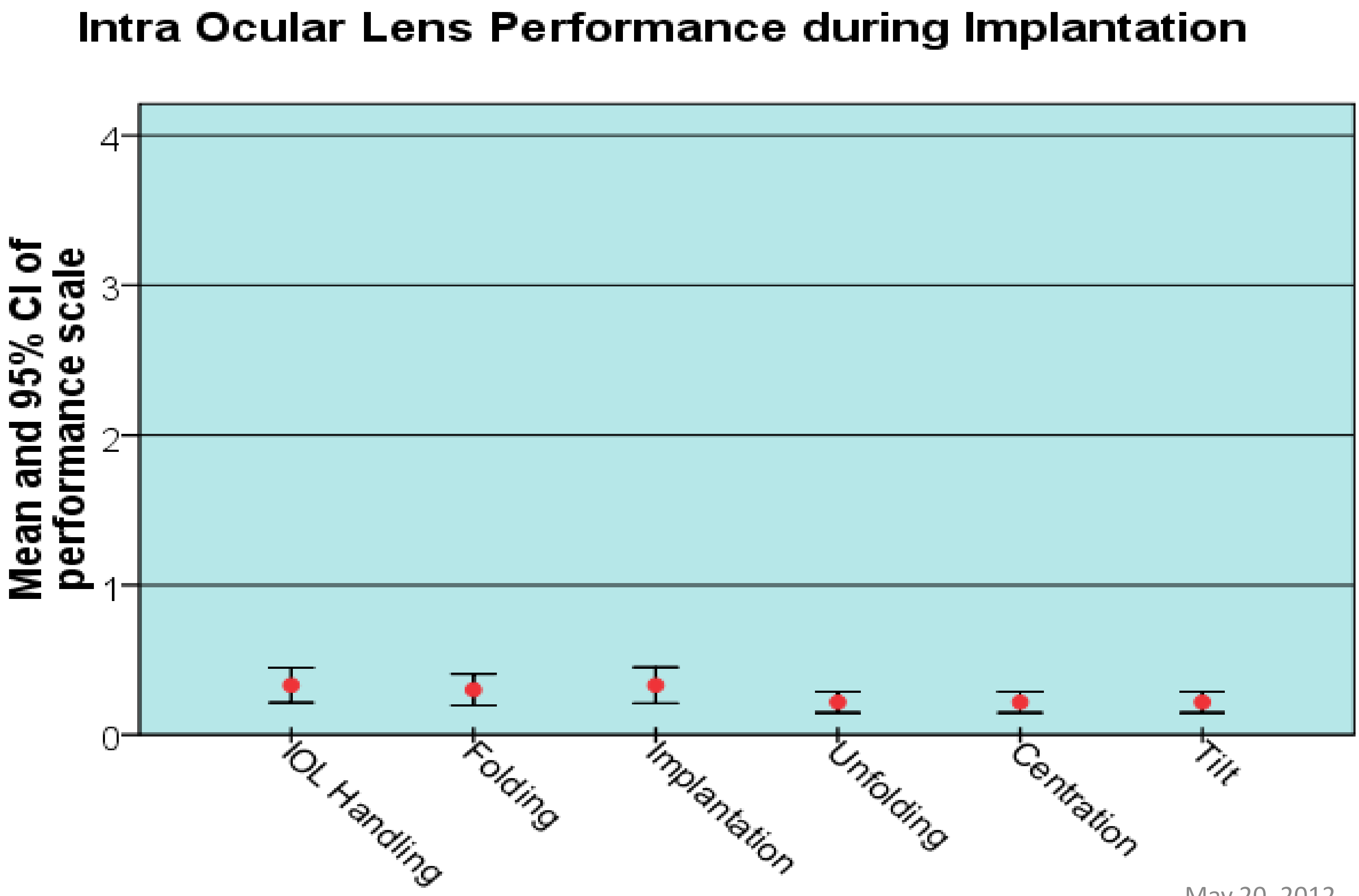

SeeLens Clinical Evaluation

Tilt & Decentration

Hanita Lenses

Scale : 0(Excellent performance)-4(poor performance)

All surgeons scaled the Tilt and Decentration on a 0-4 scale.
All of the lenses were reported to have ZERO Decentration & Tilt

Typical Pentacam image of the SeeLens position. by Prof. Novak

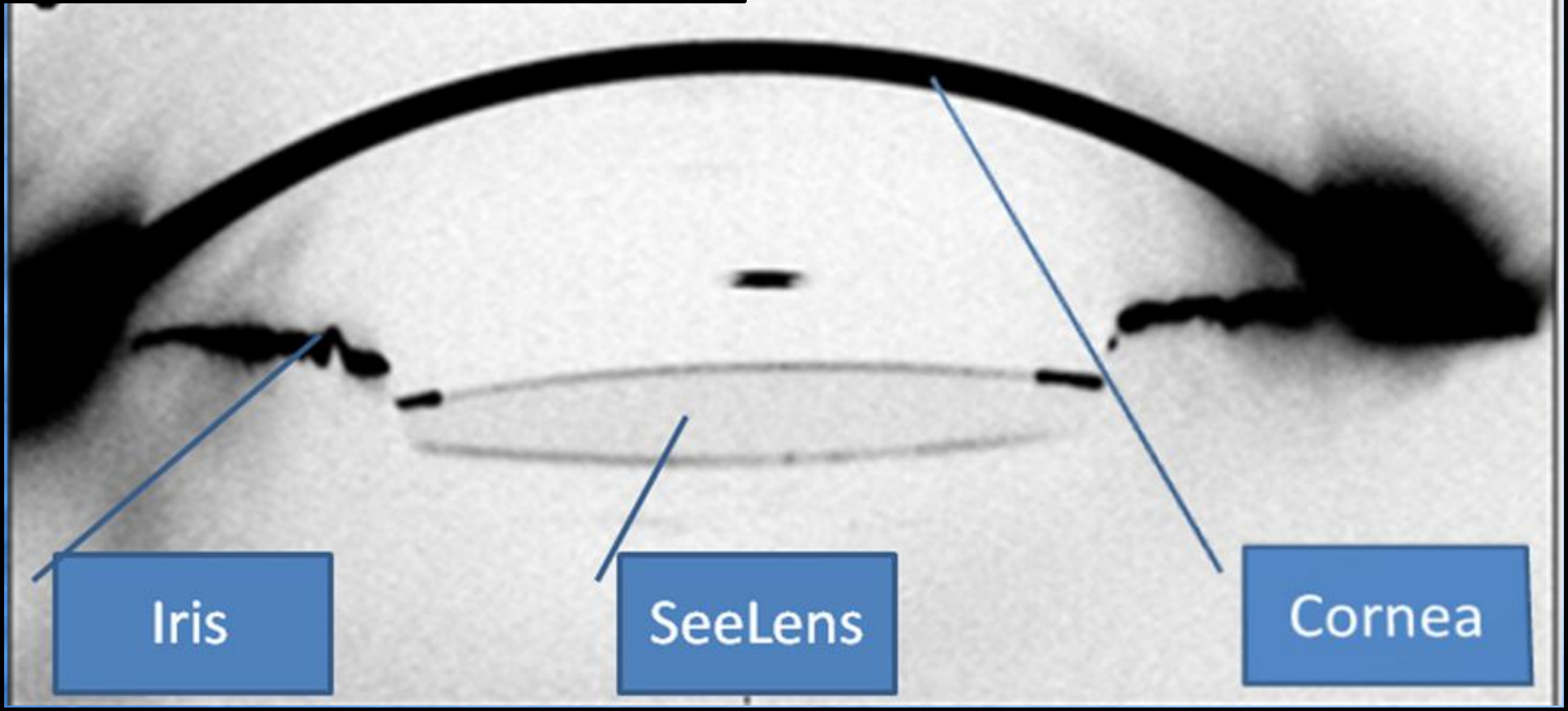

BCVA

Very good visual acuity obtained post operatively.

This rise (mean±SD: 0.49±0.04) was found to be highly significant (Paired samples t-test, $p<0.001$). The 95% Confidence Interval of the rise was 0.53 – 0.45.

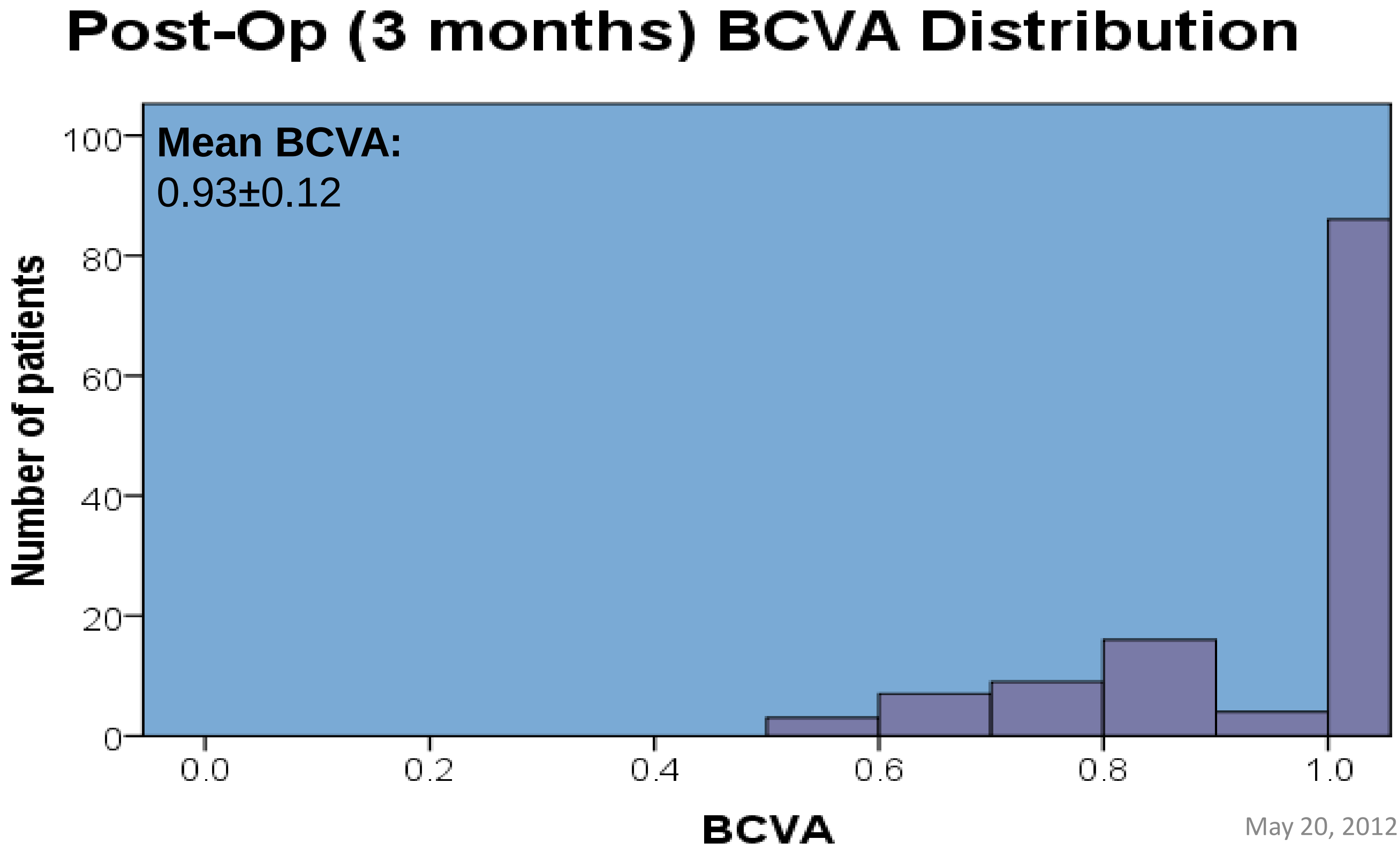

SeeLens Clinical Evaluation

BCVA

ISO Compliance

Hanita Lenses

ISO standard required to have **88%** of the patients above the 6/12 BCVA

The SeeLens achieved **100%** of the patients above the 6/12 BCVA for all patients

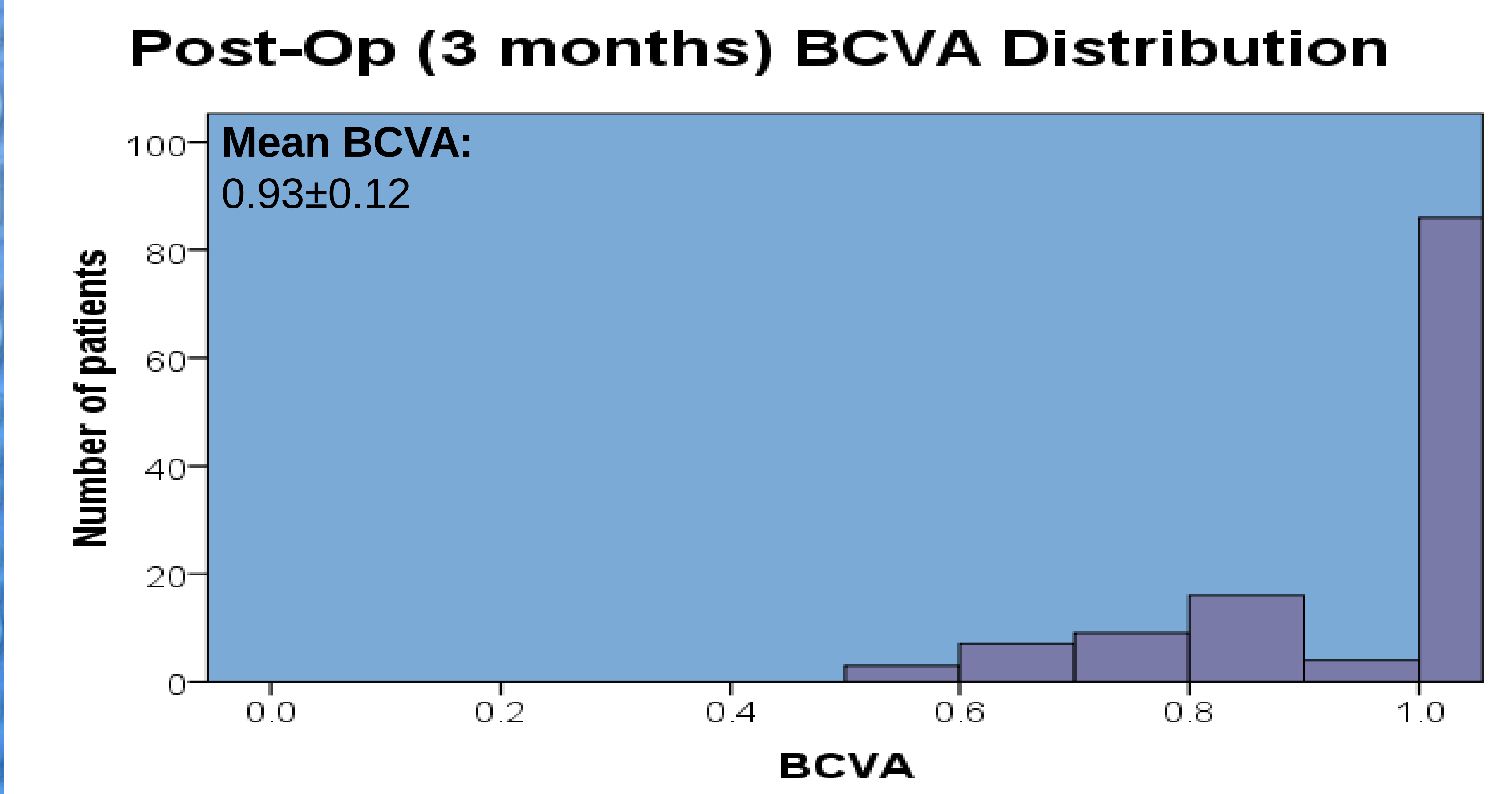

BCVA- ANOVA

ANOVA tests between the surgeons were made – finding that:

❖Differences between 2 surgeons for BCVA Pre Op were significantly different – surgeon 1&4.

❖The significant difference between the groups has remained also in the post op values.

Pre &Post op mean BCVA per surgeon

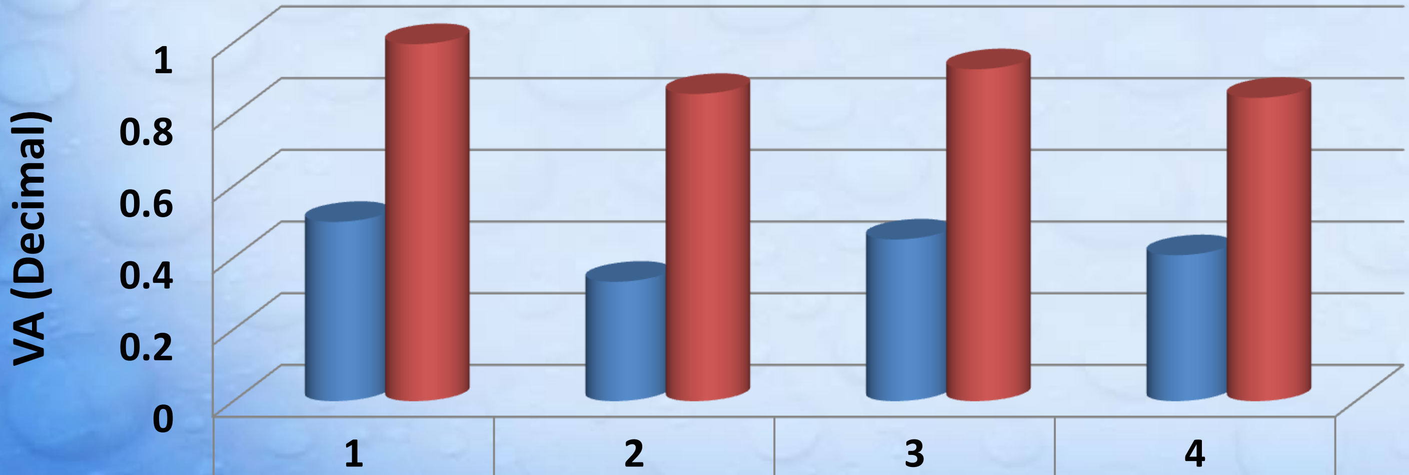

	1	2	3	4
Preop	0.5	0.333	0.451	0.407
post op	0.996	0.857	0.926	0.846

SeeLens Clinical Evaluation

Diopter Shift

Hanita Lenses

- The diopter shift deviates around the center.
- All refraction calculations were conducted using an estimated A-constant of 118.6 in the Zeiss IOLMaster SRK/T formulation.
- The mean targeted refraction difference (diopter shift) is -0.14D

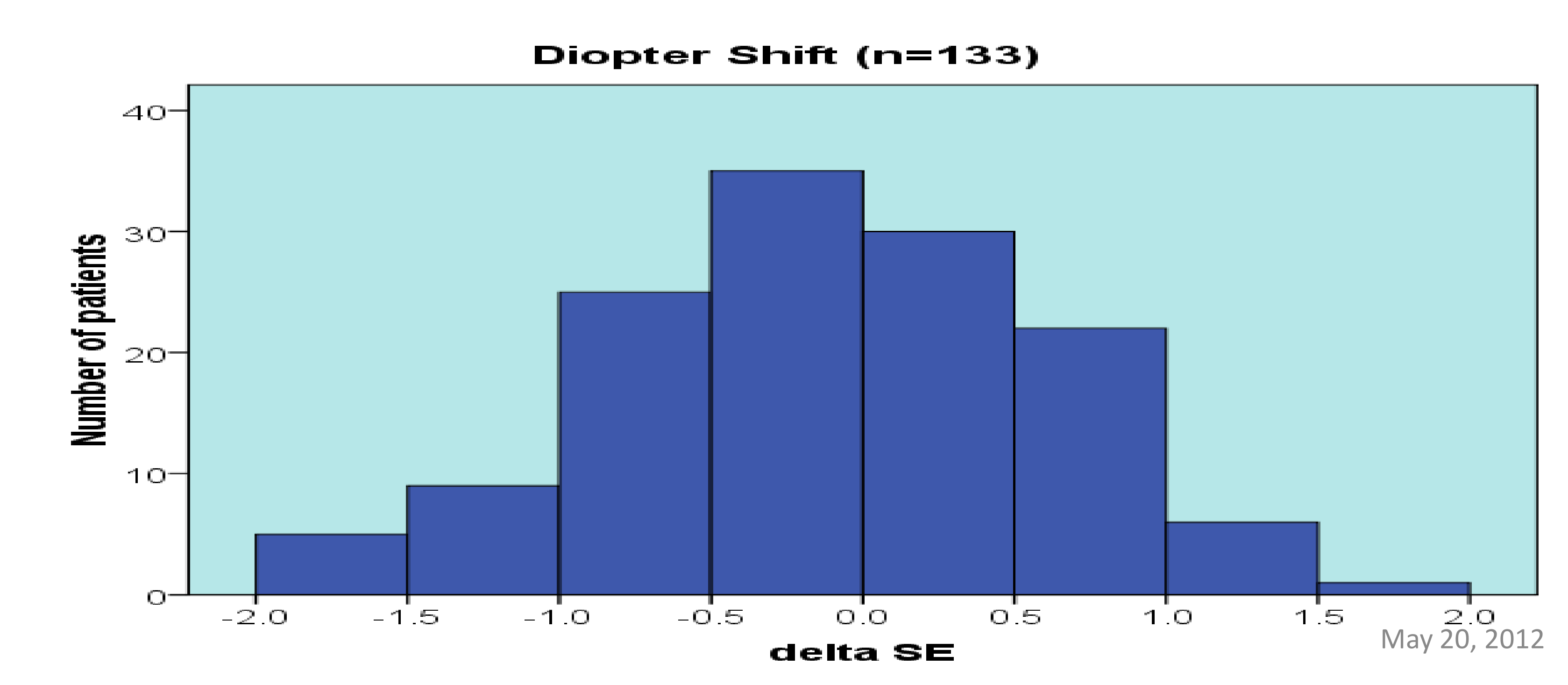

SeeLens Clinical Evaluation

Diopter Shift

- The extreme values outside the range of (+1)~(-1) occurred mainly in one surgeon with very low targeted refraction the rest show low SNR in the IOLMASTER reading

From the OSN quoting a study in sweeden of 23,244 eyes:

***J Cataract Refract Surg*. 2008;34(11):1935-1939.**

"19,489 patients (83.8%) were within ±1 D of the difference between targeted refraction and postop refraction."

In the SeeLens study 84.2% of the patients were within the middle ±1 diopter shift range.

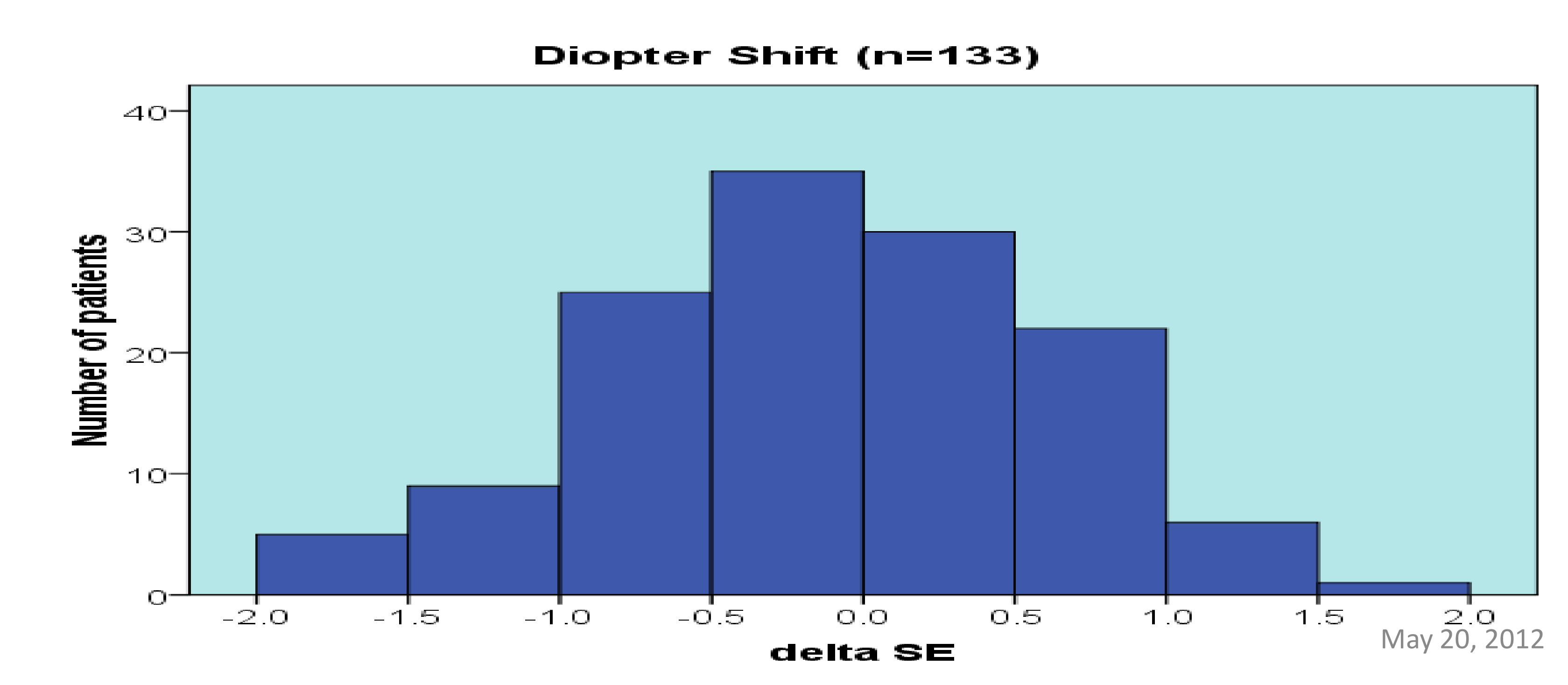

Operative and measured data

Hanita Lenses

Axial length: 23.45±0.85[mm], range 21.69[mm]~25.83[mm]

Averaged K reading K=44.1±1.4 [D] ranged 40.47[D] ~47.21[D]

Mean diopter power used in the operations: 21.12±2.03D, range15D to 25D

Mean incision size before implantation: 2.62±0.42 mm

Mean incision size after implantation: 2.68±0.32 mm

Mean post op spherical equivalence was calculated to be : -0.519D

Targeted refraction: -0.4±0.464[D] ranged +0.2[D]~ -3.0[D]

SeeLens Clinical Evaluation

Intraocular Pressure

As can be noticed 100% of the eyes IOP remained within the normal range of IOP with in population.
No high intra ocular pressure was induced to the eye after the implantation with the SeeLens IOL

Pre-operative IOP was 14.98±2.61 mmHg,
Post –operative IOP was 13.18±2.66 mmHg.
This decrease (mean±SD: 1.89±2.95 mmHg) was found to be statistically significant (Paired samples t-test, $p<0.001$). The 95% CI of the decrease was 1.366-2.410 mmHg).

Two patients of the same surgeon were treated by YAG laser 1month post op due to the remains of a cohesive adhesive Ophthalmic Viscoelastic Device (OVD) in the posterior capsule.
Both patients achieved normal IOP and 6/6 visual acuity.

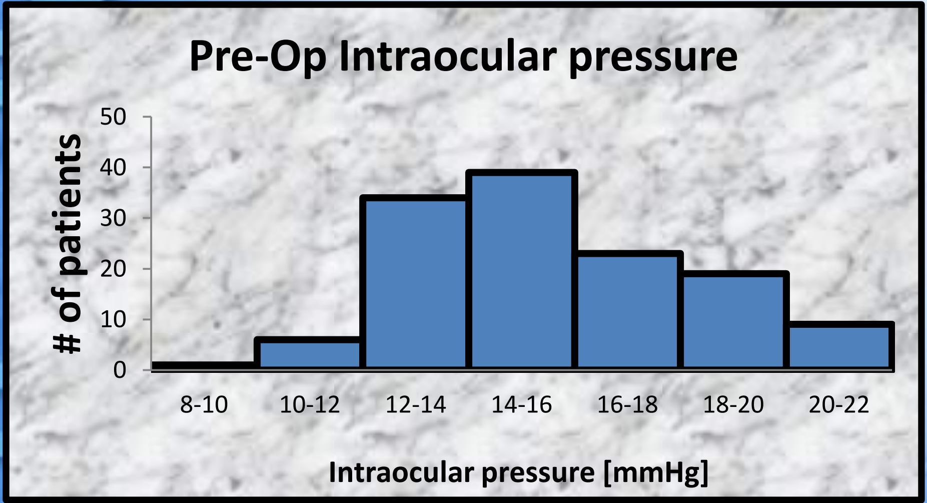

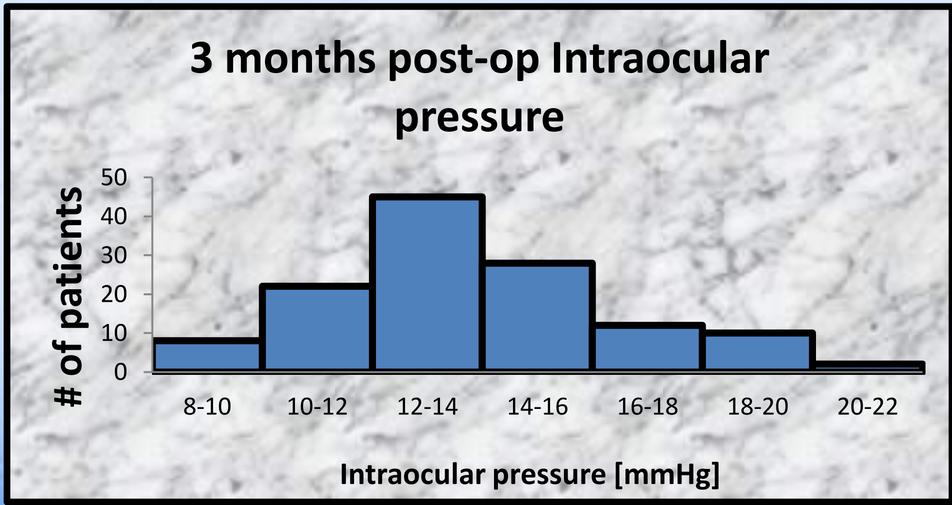

SeeLens Clinical Evaluation

Damage to IOL

Out of 133 implantations 5 damages to IOL occurred:
2 scratches on the periphery of the optic – IOLs not replaced, no complaint from patient.
2 scratches occurred on the haptic- IOLs were not replaced, lens stable in the capsule.
1 haptic tore because of bad placement in the cartridge- lens was replaced.

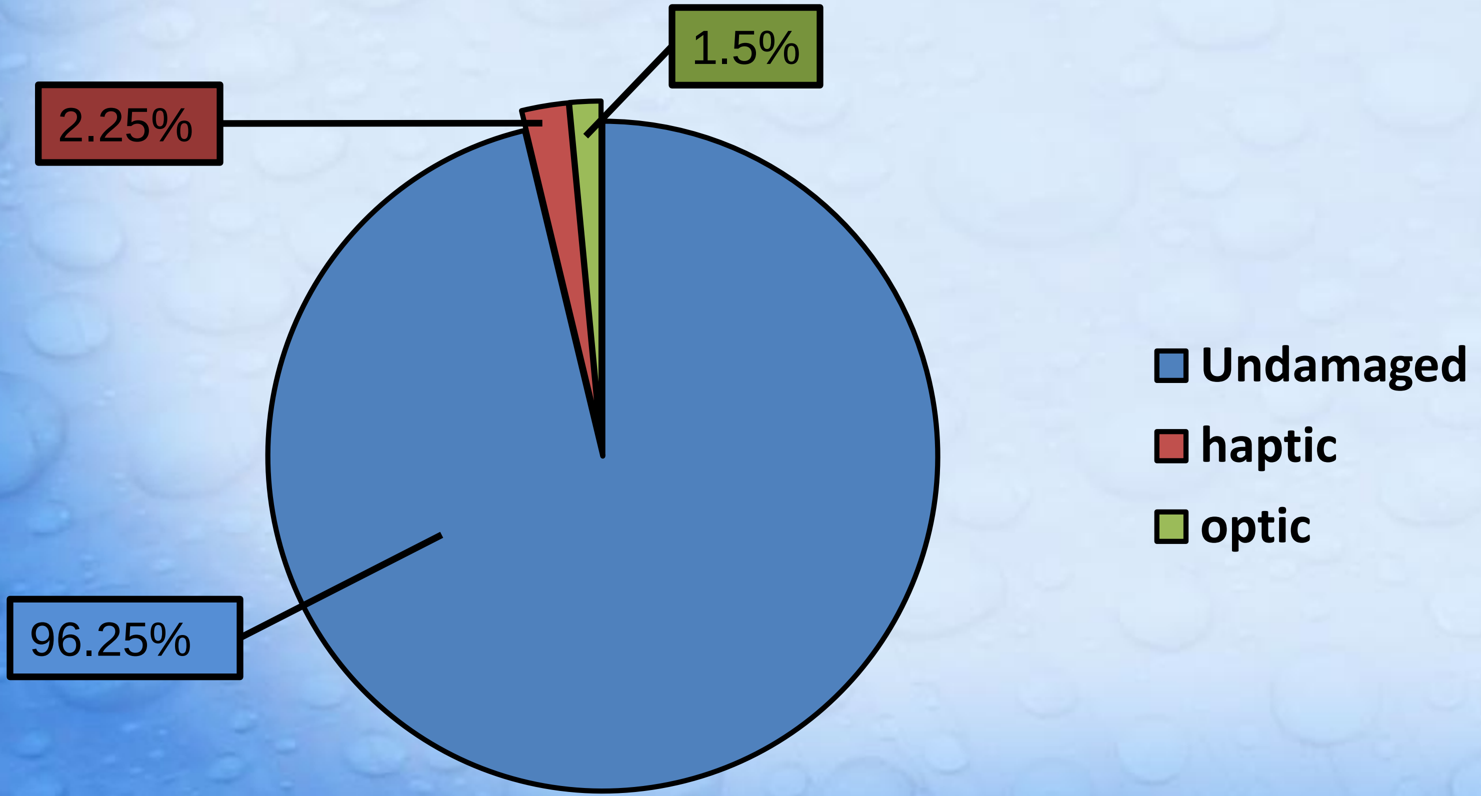

Postoperative complication

Postoperative complications, such as irritated Conjuctiva, flared anterior chamber, Corneal Edema, etc., were noted and reported by the surgeons, as requested by the protocol.

There was no evidence of post operative infection or excessive inflammation in any of the patients.

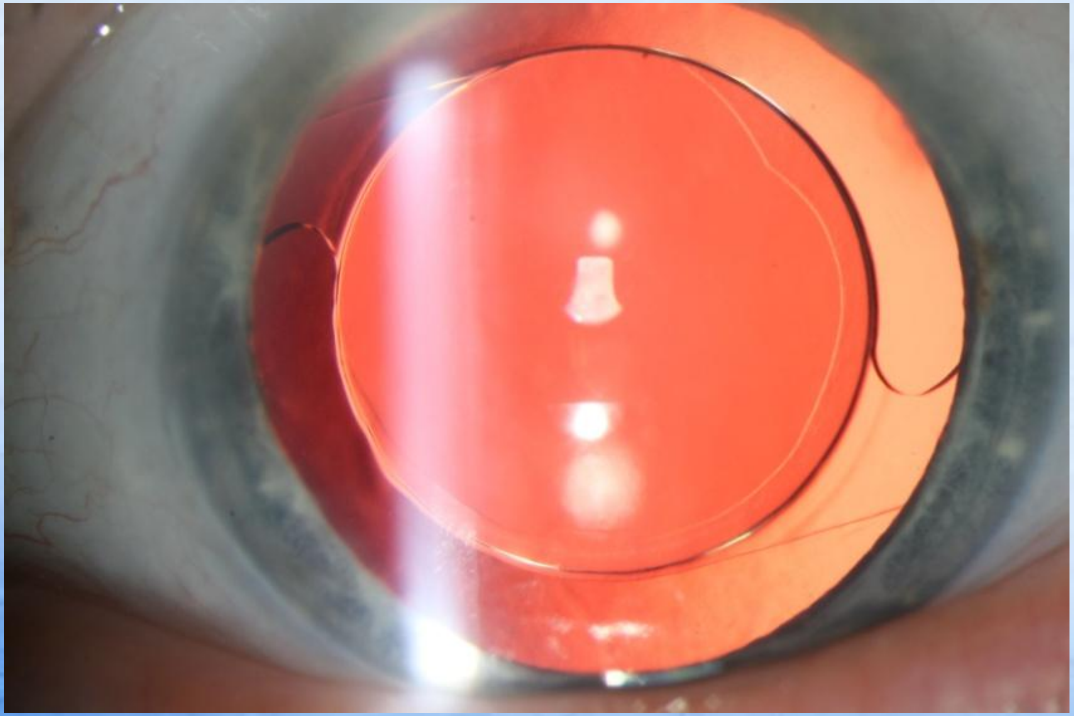

Conclusions – 3 month FU

Hanita Lenses

✓ **BCVA**
All patients achieved above 6/12 BCVA
70% achieved 6/6 vision,

✓ **Predictability**
Diopter shift is well centered around the zero value.
50% of the patients were corrected to their targeted refraction.
It is clear that the A-constant value is stable on118.6.

✓ **Behavior during implatation**
On a scale of 0-4 the lens was rated between 0 to 1 at all parameters

✓ **Infection or inflammatory reaction**
No infection or inflammatory reaction was reported.

✓ **PCO**
Until now no case of PCO was recorded.
Will be investigated in the 1&2 year follow up.
1 year follow up has started on November 2008.

✓ **Compliance to ISO standard**
The SeeLens evaluation results until now completely comply to the ISO 11979-7 2001

SeeLens Clinical Evaluation

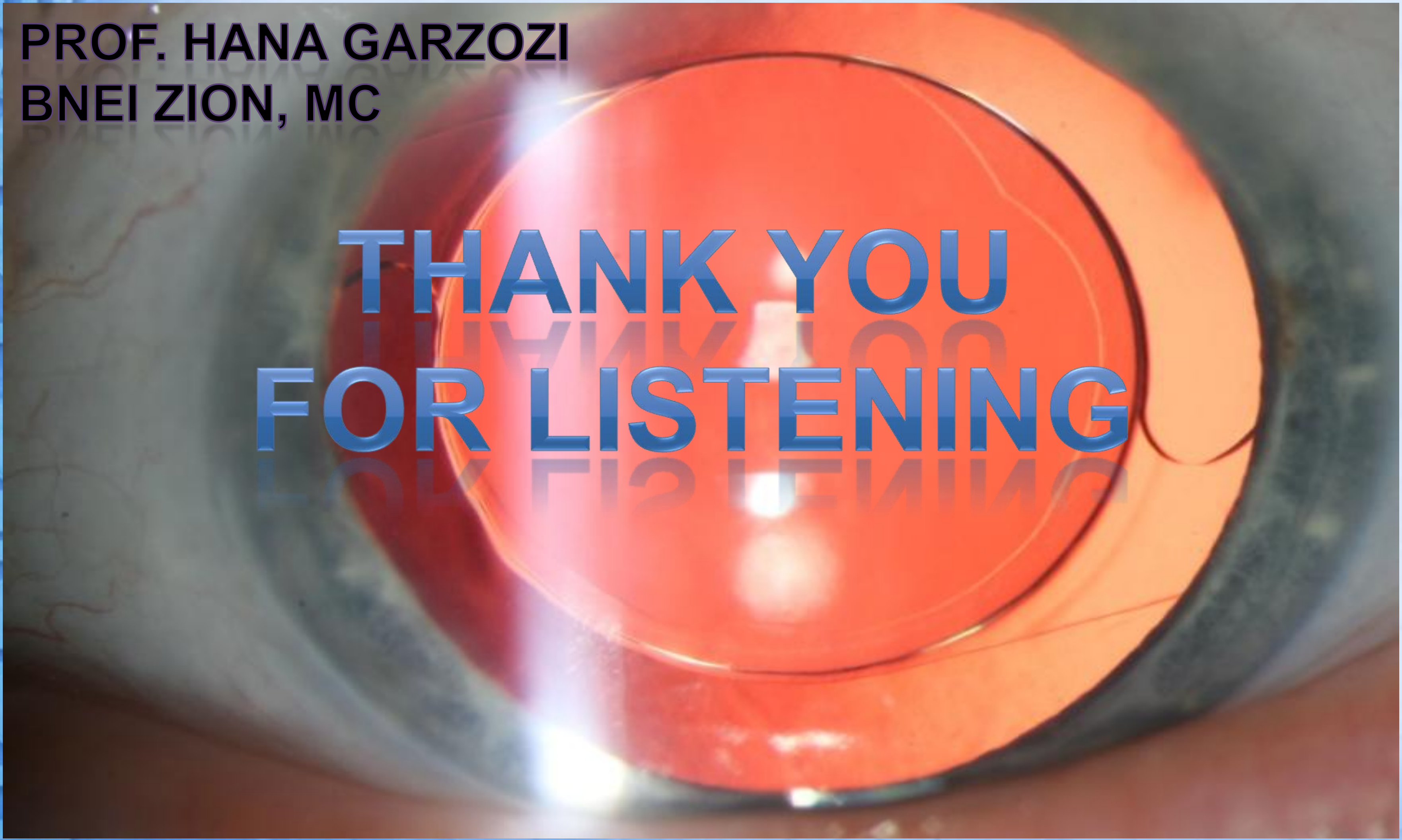

PROF. HANA GARZOZI
BNEI ZION, MC

THANK YOU FOR LISTENING
