# Supplementary material for: Intermediate term follow-up after a single-piece-acrylic intraocular lens implantation in the ciliary sulcus- a cross-sectional study
Source: BMC Ophthalmol. 2013 Dec 9;13:76. doi: 10.1186/1471-2415-13-76 (PMC4098571; doi:10.1186/1471-2415-13-76)
Supplement: Additional file 2 — SeeLens intraocular lens clinical evaluation. Description of data: an official publication of Hanita Lenses reporting clinical evaluation of SeeLens intraocular lens after 2-year follow-up, published in April 2010. [file 1471-2415-13-76-S2.pdf]

עדשות חניתה
Hanita Lenses

Clinical Evaluation 2-year Follow-up Report

of SeeLens IOL

April 2010

Table of Contents:

Objectives			2
Medical device specification and administration			3
Methods			4
Protocol of the study			6
Results			9
	a.	IOL performance during implantation	7
	b.	IOL position: Centration and Tilt	9
	c.	Post-operative Best Corrected Visual Acuity	10
	d.	Intraocular Pressure Changes	12
	e.	Posterior capsule opacification rate	13
	f.	Intra-operative complications	14
	g.	Postoperative Complications	16
	h.	Adverse Events	17
Conclusions			18

Objectives:

The objective of this study is to evaluate the safety and efficacy of the SeeLens IOL, implanted following cataract removal by phacoemulsification.

1. The key efficacy parameters are:
    - Best Corrected Visual Acuity (BCVA)
2. The key safety parameters include:
    - IOL behavior during implantation and follow-up
    - IOL related infection and/or inflammatory reactions
    - Posterior Capsular Opacification (PCO)

Efficacy and safety Assessments

The efficacy and safety assessments were determined as defined by and according to the ISO 11979 directive. The following are the demands required by the directive:

1. Post Operative BCVA of at least 6/12 (20/40) within 88% of patients' population. For the "best cases" patients, BCVA 6/12 (20/40) or better, for at least 94% of the patients. (Requirements defined by ISO 11979-7 2001 for a sample size of 100 patients).

2. IOL related Post Operative complication and Adverse Events equal to or less then the allowed rate defined by ISO 11979-7 2001.

Medical Device Specification and administration:

Specifications:

SeeLens is a foldable Hydrophilic Acrylic IOL with a C-loop haptic design and a double square edge.
The lens design offers many advantages in terms of:
1. C-loop haptics designed in order to absorb pressures and compression, which ensure centricity and long-term stability.
2. Predictability of refractive correction.
3. Very low rate of posterior Capsular Opacification – Double square edge stepped barrier, designed to
 reduce PCO (helps prevent cell migration onto the posterior capsule).
4. Ease of injection through a 2.4mm injector cartridge
5. Excellent memory – slow gentle release, superior foldability – slow and gentle unfolding.

**Overall length**	**13.0 mm**
**Optic Diameter**	**6.0 mm**
**Power range**	**+1 to +7 & +31.0 to +40.0 (1.0 increments)**<br>**+8 to +30 (0.5 increments)**
**Optic design**	**Equi -convex**
**Lens design**	**Double square edge with stepped barrier**
**Haptic angulation**	**5°**
**Material**	**Hydrophilic Acrylic UV blocker**
**Refractive index**	**1.462 (35° c)**
**YAG laser**	**Compatible**
**A constant**	**118.6**
**Placement**	**Capsular bag or Sulcus**

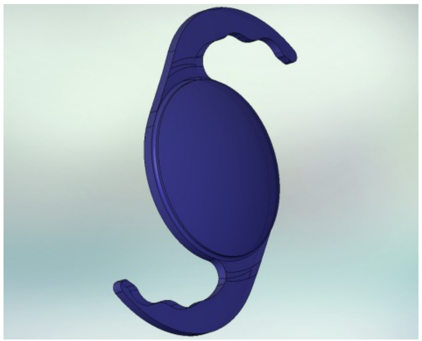

Administration:

The SeeLens IOL is administrated into the capsular bag by an Intra Ocular Implantation.
Preparation stages include lens folding into a cartridge and charging the loaded cartridge onto an injector.

Methods:

The study is a prospective, open, non-randomized, multi-center study.

Sample size: a multi-center study of 150 patients, who meet the inclusion / exclusion criteria for the study protocol across 4 centers.
The study was carried out starting from November 2008.
Patients Enrollment period: up to 2 years.

The Investigative sites are:

	Surgeon, Site	Number of implanted eyes	Number of eyes 3 months follow up	Number of eyes 1 year follow up	Number of eyes 2 years follow up
1	Prof. E. Assia<br>*Ein Tal Clinic, Tel - Aviv*	30	22	27	24
2	Prof. H. Garzozi<br>*Bnei Zion Hospital Haifa*	50	9	24	0*
3	Dr. D. Zachs<br>*Private clinic Petach Tiqwa*	20	5	11	0*
4	Dr. J. Novak<br>*Regional Hospital Pradubice Czech republic*	50	50	0**	0**
	Total	150	86	62	24

* Follow up of 1 year only was planned
** Follow up of 3 months only was planned

Statistical methods

We used the following analyses to describe the data in this report:

1. Descriptive statistics: continuous variables are described with mean ± standard deviation (SD), median, minimum and maximum and 95% confidence interval for population mean. Nominal scale variables are described with absolute and relative (percents) frequencies. Ordinal variables are described with means ± SD and frequencies of the ordinal grades.

2. All analyses were done with Excel 2007 and SPSS 15.0.

Eligibility

Subjects who met the following criteria are eligible for the study.

Inclusion Criteria

1. Age > 50 years
2. Senile cataract
3. Refractive cylinder < -1.5 diopter
4. Planed surgery technique: cataract extraction by ECCE Phacoemolsification.
5. Calculated IOL powers are in the range of 15 - 25.
   The IOL power required were calculated by the biometry measurements of the IOLMASTER.

Exclusion Criteria

1. Allergy or intolerance to required study medications (including antibiotic).
2. Amblyopia.
3. History or evidence of any Ocular disease that may effect Visual Acuity (i.e.: Uncontrolled glaucoma, Ocular injury, Corneal pathologies, Retinal pathologies in general and macular pathologies in particular, diabetic Retinopathy, Uveitis, Aniridia or iris atrophy, Vitreous pathologies (patients with vitreous separation or floaters can be included).
4. Rubella cataract.
5. Any other ocular condition that may predispose a subject to future complications or contraindicate implantation of the model SEELENS
6. Previous ocular surgery, including refraction surgery (study eye).
7. Microphthalmos.
8. Extreme axial length (outside the range of 21.5 – 25).
9. Surgical complication (not IOL related), that may effects study results (i.e. Capsular tear or rupture during cataract extraction).
10. Posterior Capsule scar or opacification if demonstrated during the cataract extraction surgery.
11. Multiple operative procedures during cataract extraction.

Withdrawal Criteria:

Patient' data was not included in the analysis at the following conditions:

1. Patient is not complying with the follow up visits.
2. During the study period, patient developed systemic disease that may affect vision (i.e.: CVA).
3. During the study period, ocular disease that may affects vision and is not IOL related had occurred (i.e.: ocular injury).

IOL exchange:

IOL extraction was allowed only by the indication of one or more of the following:

1. Refraction deviation entails IOL extraction (upon clinical judgment).
2. Haptics' tear that jeopardized IOL stability and centration.
3. Persistent sever infection required IOL extraction (upon clinical judgment).
4. IOL opacification which entails IOL extraction (upon clinical judgment).

Protocol of the study:

Pre-operation tests and measurements

The following routine measurements recording of patients with senile cataracts who were candidates for cataract extraction with IOL implantation:

- Medical History and demographic data.
- Visual Acuity (BCVA, UCVA).
- Refraction.
- Keratometry.
- Axial Length
- IOL power selection.
- Intraocular Pressure measurement
- Slit lamp examination (Conjunctiva, Cornea, Anterior Chamber, Iris, Pupil, Fundus).

Eligible patients were enrolled at the study.

Operation Procedure:

The study Surgery Procedure: cataract extraction by ECCE Phacoemolsification. Operation was performed according to the routine surgery technique of the investigative sites.

Post Operative Reports:
The following Parameters were recorded at the end of each Operation:
- Operation Procedures data
- Surgical complications.
- Ease of IOL Implantation.
- IOL location and centeration.
- Posterior Capsule abnormal condition (i.e. scar, tear etc.)

Study Visits:
Specific study visits were required at 1 day, 7-10 days, 2-3 months postoperatively, and follow up study visits will be conducted at 1 year and 2 years periods. Postoperative procedures at each visit will include the following measurements:
- Uncorrected and Best-corrected visual acuity (except for day 1)
- Manifest refraction.
- Intraocular pressure.
- Slit lamp examination (The same tests performed preoperatively)
- IOL location and centration.
- Records of Post operative complication and
- Adverse Events (see appendix 1)
- Extent of PCO (see appendix 1)

Results:

a. IOL performance during implantation

Implantation parameters were measured to indicate the performance of the SeeLens IOL implantation during the standard cataract surgery. The implantation parameters that were measured during the implantation were SeeLens' handling, folding, implantation, unfolding, need to manipulate the lens and the final centration and tilt of the lens position at the end of the procedure.
All parameters were graded on a 0 (best) to 4 (worst) scale.
See protocol for more details.
The results are presented in Graph no. 1

Graph 1: IOL performance during implantation (implantation parameters):

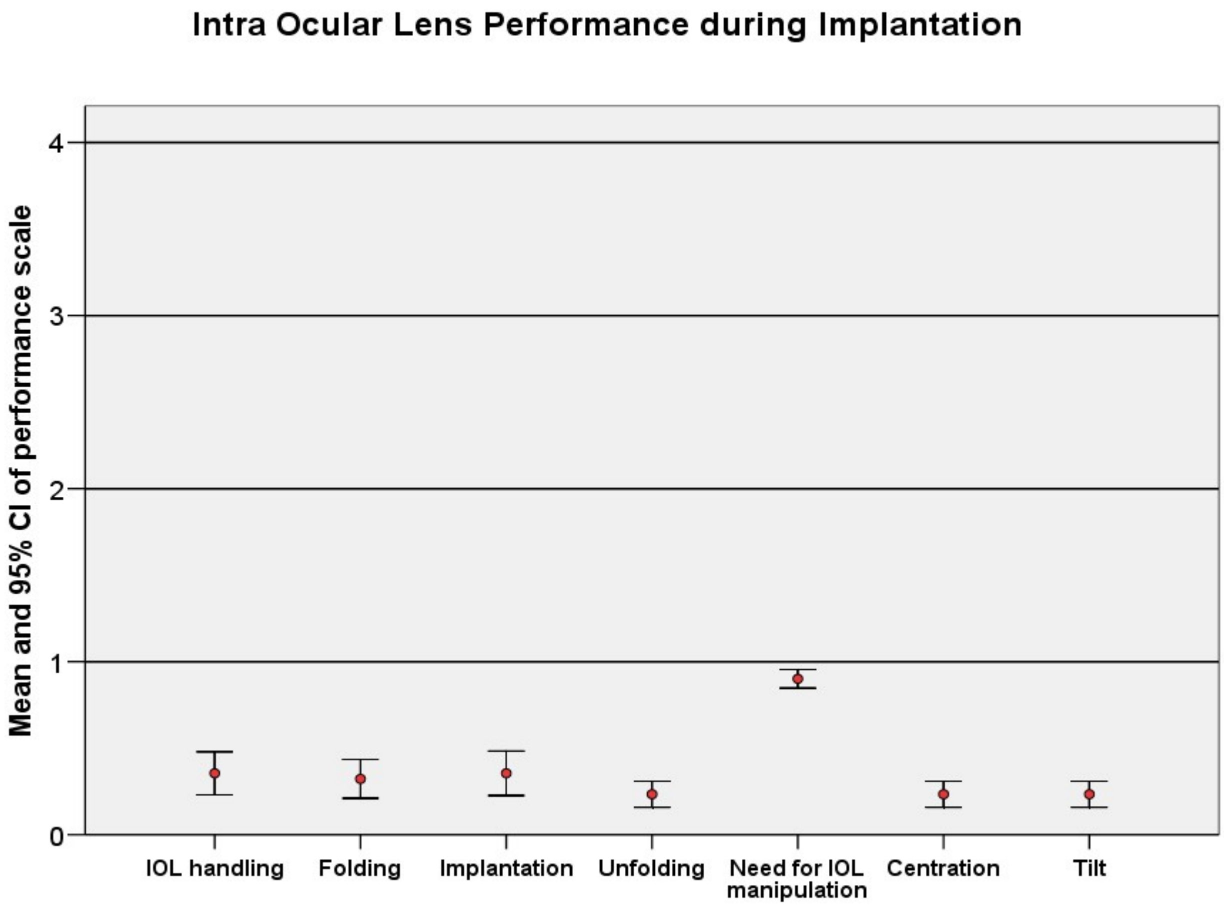

As can be seen on graph 1, all surgeons reported that the SeeLens handling, folding and injection is easy and smooth. A few complications were reported:

1. In one case an iris hook had to be used since pupil did not dilate
2. In one case an opening at the posterior capsule was noticed
3. In a trial use of a bevel up cartridge (a newly developed device), the lens got into the bag upside down, and it was required to rotate the lens to establish its normal position.

None of the surgical complications described above was caused by the SeeLens intra ocular lens, and none caused any damage to the visual acuity of the patient.

b. IOL location and centration

Lens location and centration were noted and reported in all follow-up visits. Centration and tilt were graded on a 0 (best) to 4 (worst) scale. See protocol for more details.

Image1 : Oculos Pentacam image of the SeeLens in the eye 3 month post op

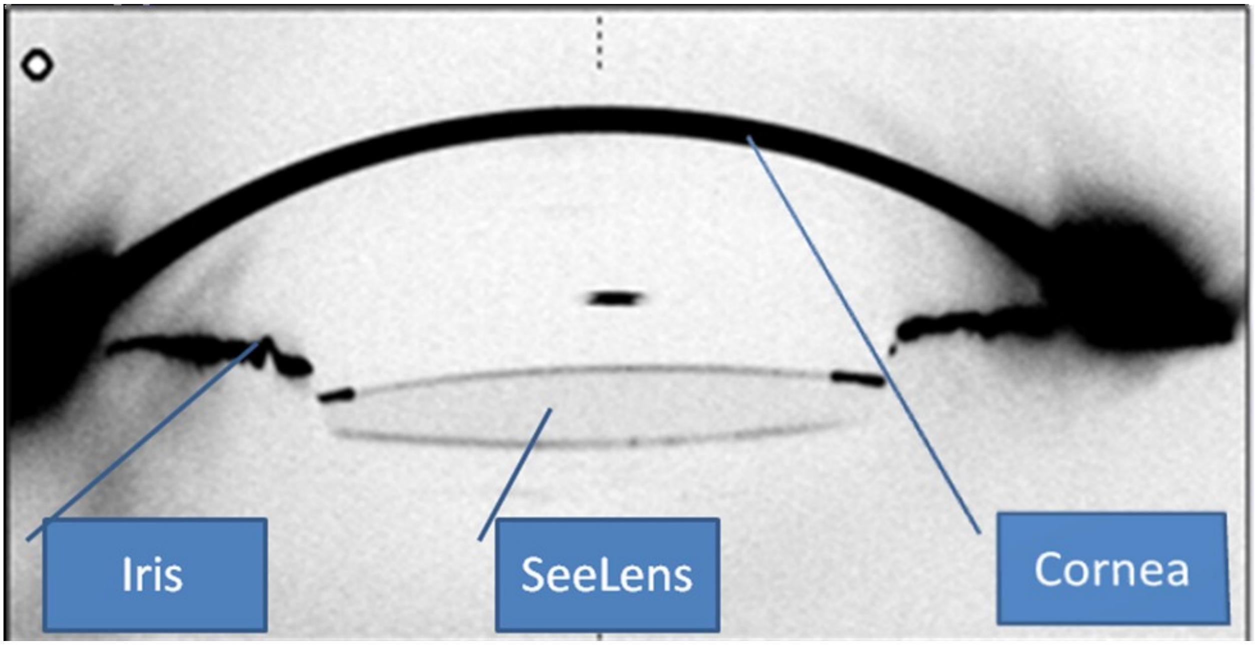

Image 1 demonstrates a typical image of the SeeLens IOL position 3 months post op position. It can be inferred from image 1 that the SeeLens is both well centered and with no tilt.

All lenses during follow up visits including 2 years follow-up were reported as zero (Best) centration and tilt.

Therefore the SeeLens demonstrates good stability and centration from implantation throughout the 2 years follow-up period.

c. Postoperative Best Corrected Visual Acuity (BCVA)

Best Corrected Visual Acuity (BCVA) was reported in the pre-operative and the post-operative follow-up visit. Pre-operative BCVA results are shown on Graph 2.

Graph 2: Pre-operative BCVA distribution

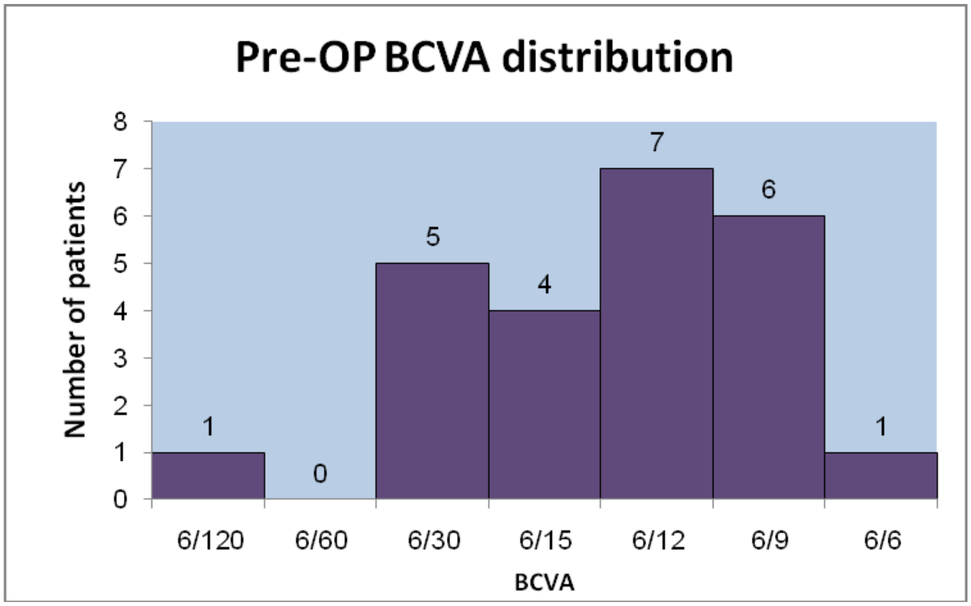

Post-operative BCVA results are shown on Graph 3.

Graph 3: 2 years Post-operative BCVA distribution

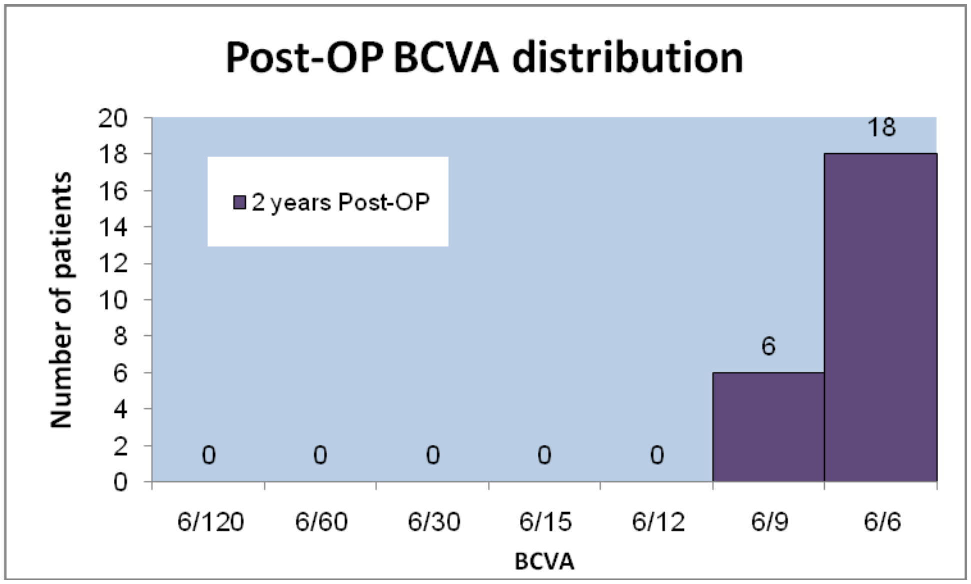

Pre- and post-operative BCVA are compared in the following tables 1 and 2.

Table 1: Pre-operative BCVA

	% of patients
6/12 or better	58.3%
6/15 or better	75%
6/30 or better	95.8%
Worse than 6/60	4.2%

Table 2: 2 years Post-operative BCVA

	% of patients
6/6	75%
6/9 or better	100%
Worse than 6/12	None
Average VA	0.96 or 6 / 6.3

As shown in table 2, postoperative (2 years) VA of 6/9 or better was reported in 100% of all eyes.

Statistical comparison between Pre- and post- operative BCVA:
Prior to surgery, the mean BCVA was 0.46 (or 6/13.3), and 2 years after surgery it averaged 0.987 or 6 / 6.07.

Thus, it can be concluded that the SeeLens provides excellent optical performances as shown above, and is fully in accordance to the ISO 11979-7 2001 as noted in the Efficacy and Safety Assessment criteria 1.

d. Intraocular Pressure Changes:

Changes in intraocular pressure (IOP) from the pre-operative to 2 years postoperative visits are shown in graphs 5-6 below.

Graph 5: Pre-operative IOP pressure

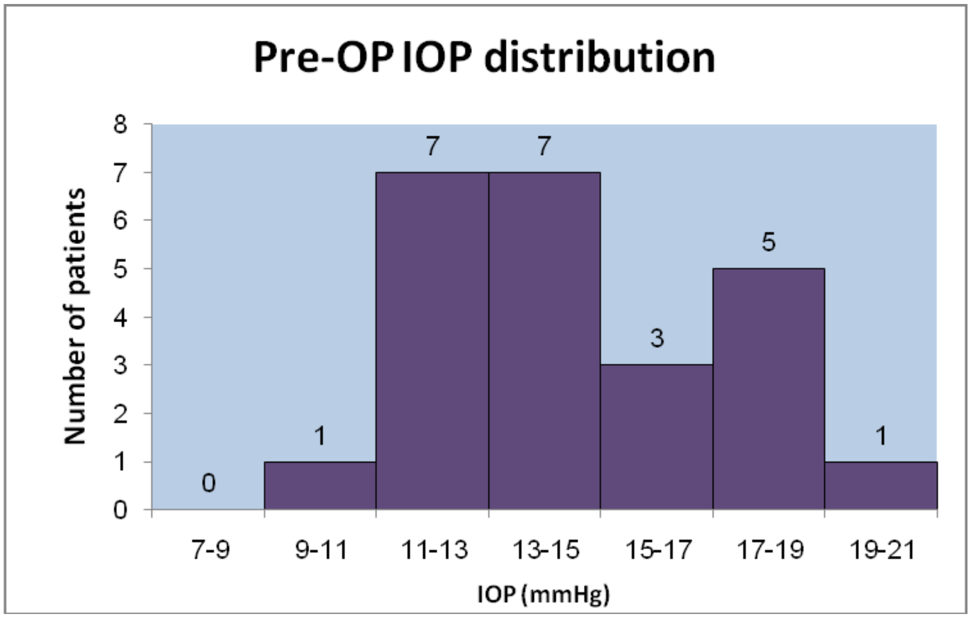

Graph 6: 2 years Post-operative IOP pressure

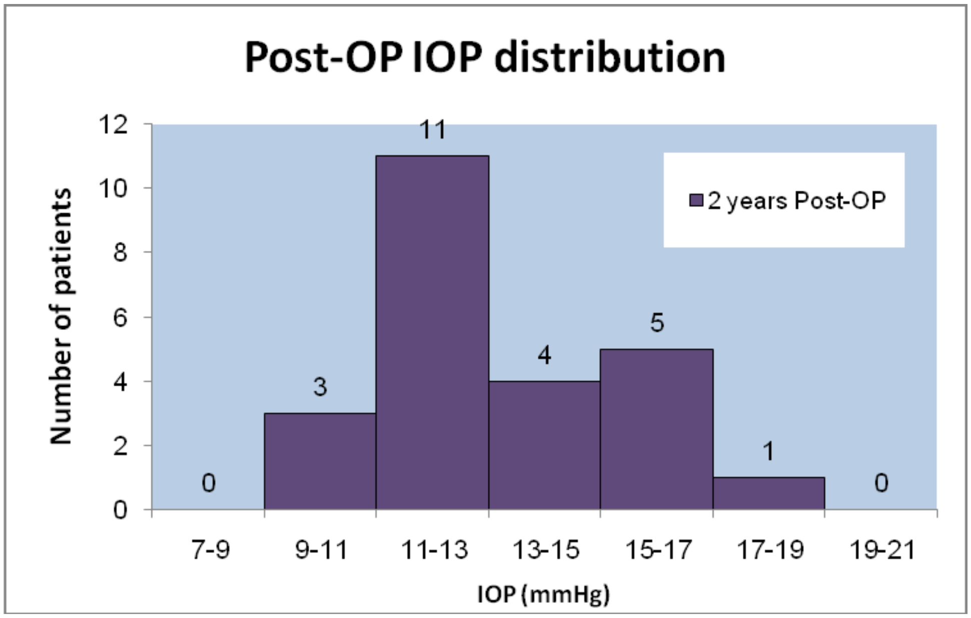

As can be noticed 100% of the eyes IOP remained within the normal range of IOP with in population. No high intra ocular pressure was induced to the eye after the implantation with the SeeLens IOL.

Similarly to reported clinical studies conducted by surgeons, a decrease of the intra ocular pressure was observed in the comparison of the IOP Pre op to Post op.
Pre-operative IOP was 15±2.7 mmHg, three months after the operation IOP was 12.6±3.3 mmHg, 2 years after the operation IOP was 13.83±2.16 mmHg.

e. Posterior capsular opacification in 2 years post operative follow up

1. The surgeons were asked to report on the PCO rate. The score scale range from grade 0 reflect non PCO, to grade 3 reflect the most severe condition.
   The results are presented in Graph no. 7

Graph 7: 2 years post operative PCO score

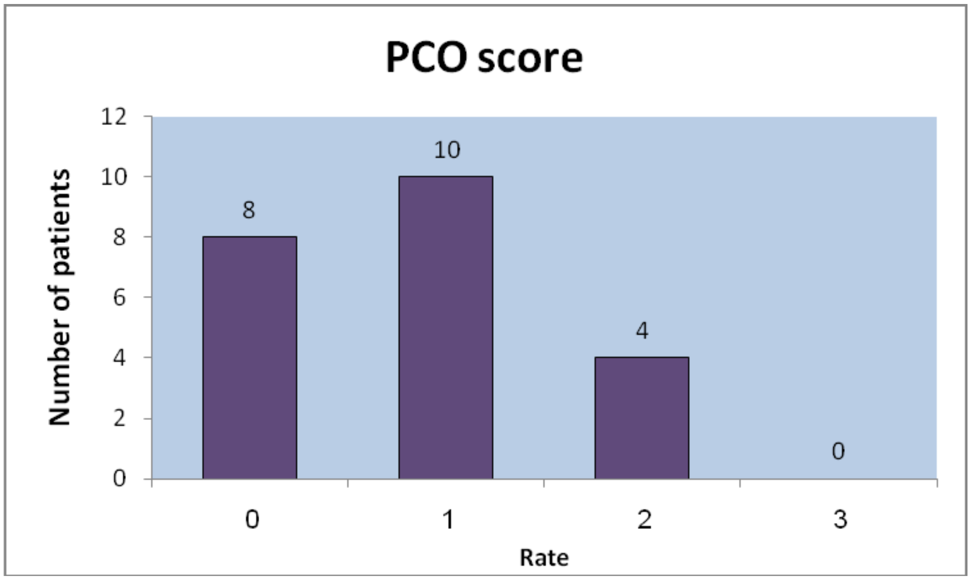

75% of implanted eyes have shown absent or mild PCO (grades 0-1). Two patients underwent YAG procedure.

f. Intra-operative complications

1. The surgeons were asked to report on need for manipulation and damage to the IOL:

Graph 8: Damage to IOL

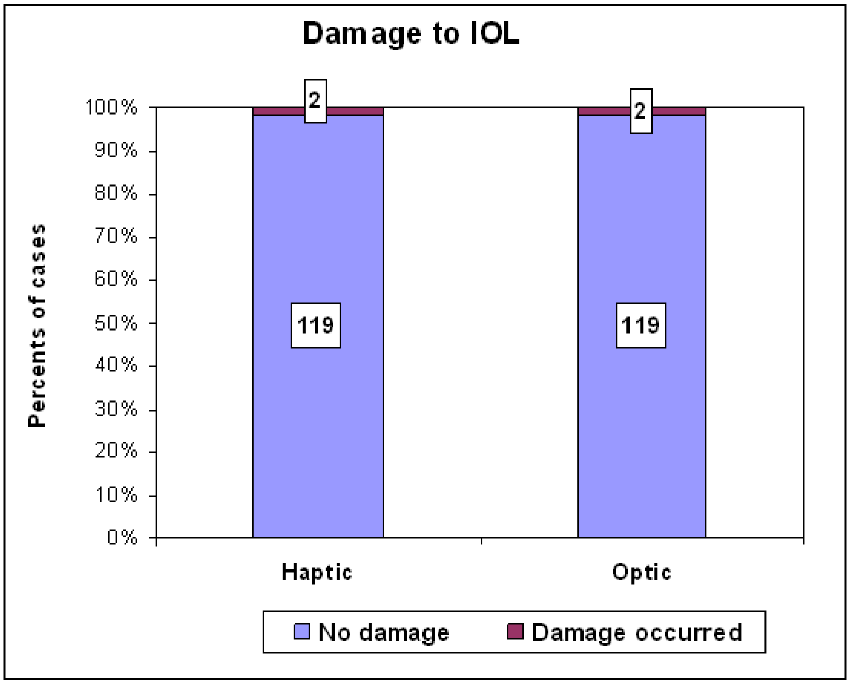

I. Optic Damage

i. In one case the lens was scratched by forceps during extraction of IOL from cartridge.
ii. Lens could not be inserted through a 2.2mm incision and was extracted and implanted once through a 2.4 mm incision. The damage to the optics was done by forceps handling while extracting the lens from the eye.

II. Haptic Damage

i. One IOL was inserted into the cartridge with one haptic outside the cartridge and the haptic broke. IOL was removed from the eye after the optic was cut with scissors. A different IOL was implanted.
ii. One IOL haptic had a few smears or scratches after implantation

In all cases the damage did not influence IOL location and centration.

2. The surgeons were also asked to report any other complication occurred during the surgery.

   i. One patient was found to be with small zonulysis. Implantation did not face any problem.
   ii. Four patients were under the influence of prostate cancer medication named OMNIC, which induced Intra Operative Floppy Iris Syndrom (IFIS). The IFIS causes the pupil to be small and thus increases the complexity of the cataract procedure.
   iii. One patient had the optics on the nasal side outside the bag, haptics are in the bag. Patients' best corrected visual acuity is 6/6.
   iv. In a trial use of a bevel up cartridge (a newly developed device), the lens got into the bag upside down, and it was required to rotate the lens to establish its normal position.
   v. In one case an Iris Hook was required,since the pupil was too small.

g. Postoperative Complications

Postoperative complications, such as irritated Conjuctiva, flared anterior chamber, Corneal Edema, etc., were noted and reported by the surgeons, as requested by the protocol.
There was no evidence of post operative infection or excessive inflammation in any of the patients.

From image 2 it can be inferred that the SeeLens is well centered. A typical clear cornea and healthy conjunctiva can be indicated, as it was seen in all SeeLens implants 3 months & 1 month post op.

Image 2: The SeeLens IOL as seen through a slit lamp

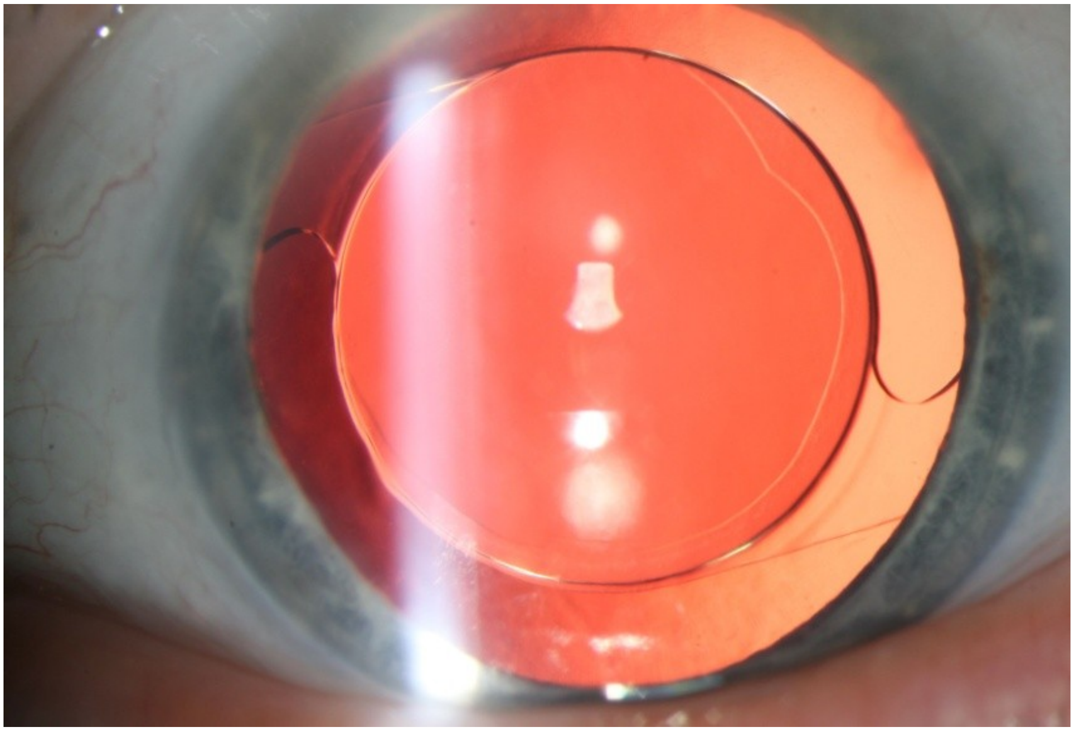

h. Adverse Events as defined by ISO 11979-7 2001

Adverse Event	Maximal allowable rate as defined by ISO 11979-7 2001 ; 100 subjects	Score / measurement					Rate of adverse effect occurred at this study	Pass/Fail
Cystoid macular oedema	6%	No / Yes					0 %	Pass
Hyphema	5%	0	1	2	3	4	0%	Pass
Hypopyon	1%	Measure in mm.					0%	Pass
Intraocular infection	1%						0%	Pass
Lens dislocation	1%	Measure in mm.					0%	Pass
Pupillary block	1%	No / Yes					0%	Pass
Retinal detachment	1%	No / Yes					0%	Pass
Secondary Surgical intervention (eccluding retinal detachment and posterior capsulotomy)	2%	No / Yes, specify					0%	Pass
Persistent								
Corneal stroma oedema	1%	0	1	2	3	4	0%	Pass
Cystoid macular oedema	2%	No / Yes					0%	Pass
Iritis	1%	0	1	2	3	4	0%	Pass
Raised IOP req. treatment	2%						0%	Pass

As shown in table 3, no advers event was reported in **100**% of SeeLens implanted eyes, in a sample size of 24 patients.

Thus, it can be concluded that the SeeLens's safety is fully in accordance to the ISO 11979-7 2001

This information supports the conclusions that were previously presented, and reassures that the SeeLens is a safe high quality predictable lens.

Conclusions:

The detailed data from the current study shows the benefits of the **SeeLens** IOL:

- **Excellent optical performances, in accordance to the requirements of ISO 11979-7 2001.**
- **Very good IOL behavior during implantation.**
- **Perfect IOL centration.**
- **Very good safety profile as reflected from: very low rate of inter- and post-operative complications and intraocular pressure changes at 2 years.**
- **Very low rate of Posterior capcule opacification at 2 years follow up.**
